# Analog Switching in Hexagonal Boron Nitride Memristors via Multiple Nano‐Filaments Confinement

**DOI:** 10.1002/smll.202504507

**Published:** 2025-06-16

**Authors:** Jaesub Song, Seokho Moon, Jinho Byun, Jiye Kim, Junyoung Choi, Hyunjeong Kwak, Inyong Hwang, Changuk Ji, Seyoung Kim, Jong Kyu Kim

**Affiliations:** ^1^ Department of Materials Science and Engineering POSTECH Pohang 37673 Republic of Korea

**Keywords:** analog switching memristor, filament confinement, hexagonal boron nitride, metal‐organic chemical vapor deposition, neuromorphic computing

## Abstract

Memristors have emerged as a key building block for artificial neural networks (ANNs), offering energy efficiency and high scalability for hardware‐based synaptic weight updates. As device miniaturization is crucial for enhancing memristor performance, hexagonal boron nitride (h‐BN) stands out as a promising resistive switching medium due to its excellent insulating characteristics even at an atomically thin scale. However, conventional h‐BN memristors suffer from abrupt switching behavior by uncontrollable filament formation, limiting their potential for ANN applications. Here, h‐BN‐based memristors exhibiting linear and symmetric analog switching by leveraging multiple nano‐filament confinement is presented. The geometric confinement between suspended h‐BN films and the apexes of GaN nano‐cones facilitates analog switching behavior, reducing cycle‐to‐cycle variation and ensuring stable consecutive operations. Electrical analyses reveal that analog switching behavior originates from the controlled formation of multiple nano‐filaments within the confined geometry. ANNs implemented with these nano‐filaments confined to h‐BN memristors exhibit highly linear and symmetric synaptic weight updates, enabling precise training with minimal accuracy degradation. This work establishes multiple nano‐filament confinement as a universal design strategy for achieving reliable and linear analog switching in memristors, paving the way for advanced neuromorphic computing.

## Graphical abstract

Hexagonal boron nitride memristors with suspended structures over GaN nano‐cones enable linear and symmetric analog switching through multiple nano‐filament confinement, overcoming challenges from abrupt switching. This work demonstrates reliable synaptic weight updates for artificial neural networks, with high classification accuracy in MNIST tasks, advancing neuromorphic computing systems with improved energy efficiency and stability.

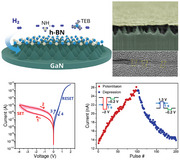

## 1 Introduction

Hexagonal boron nitride (h‐BN) is a 2D layered material with a wide bandgap^[^ ^1^ ^]^ excellent thermal conductivity,^[^ ^2^ ^]^ chemical stability,^[^ ^3^ ^]^ and electrical endurance.^[^ ^4^ ^]^ These properties have attracted widespread attention, establishing h‐BN as an ideal insulating material for 2D electronics.^[^ ^5^, ^6^, ^7^, ^8^ ^]^ Benefiting from its wide bandgap (≈6 eV) and superior in‐plane thermal conductivity (≈751 W m^−1^ K^−1^), h‐BN is particularly advantageous for resistive switching in memristors, even at atomic‐scale thickness.^[^ ^9^, ^10^, ^11^, ^12^, ^13^, ^14^, ^15^ ^]^ This enables high energy efficiency^[^ ^10^, ^11^ ^]^ and dense integration^[^ ^12^ ^]^ in synaptic crossbar arrays, which is crucial for advancing artificial neural networks (ANNs). Furthermore, recent progress in scalable, multi‐wafer growth of h‐BN via metal‐organic chemical vapor deposition (MOCVD) has enabled the fabrication of high‐quality films with wafer‐scale uniformity. This enhances its compatibility with Si CMOS technology,^[^ ^11^, ^12^, ^14^, ^16^, ^17^, ^18^ ^]^ paving the way for practical applications in next‐generation memory devices.

Memristors, considered as the simplest neuromorphic computing memory device, feature a vertically stacked two‐terminal configuration with an insulating resistive switching layer positioned between top and bottom electrodes.^[^ ^19^, ^20^, ^21^ ^]^ This straightforward design allows high‐density integration in crossbar arrays^[^ ^22^ ^]^ and ensures high‐speed operation.^[^ ^23^ ^]^ In filamentary‐type memristors, the electric field between two electrodes induces the migration of metal ions or vacancies, leading to the formation of conductive filaments (CFs) through the resistive switching layer.^[^ ^24^ ^]^ However, the formation of large‐diameter CFs leads to abrupt transitions between the low‐resistance state (LRS) and the high‐resistance state (HRS), causing a binary conductance mode.^[^ ^25^, ^26^ ^]^ In contrast, memristors for ANN applications require multi‐level conductance, known as analog switching (i.e., gradual switching) to ensure precise operation with linear and symmetric synaptic weight updates.^[^ ^20^, ^27^ ^]^ Additionally, large CFs can cause excessive Joule heating and uncontrolled ion migration, accelerating the degradation of the resistive switching layer and compromising the long‐term stability of memristor operation.^[^ ^13^ ^]^

To achieve multi‐level conductance, transistors are commonly employed as current‐regulating elements in a 1‐transistor‐1‐memristor (1T1M) configuration, where they also serve as selectors.^[^ ^9^, ^25^, ^26^, ^28^, ^29^ ^]^ However, this approach introduces fabrication challenges, increases device complexity, and limits integration density, underscoring the need for a transistor‐free analog switching memristor. A promising alternative is the precise control of multiple nano‐filament formations within the resistive switching medium.^[^ ^30^, ^31^, ^32^, ^33^, ^34^ ^]^ The sequential development of these nano‐filaments under increasing electric fields can effectively regulate the current flow, enabling stable and incremental multi‐level conductance states. Various strategies for have been explored to induce multiple nano‐filaments, including confinement through grain boundaries,^[^ ^30^, ^35^ ^]^ nanopores,^[^ ^31^, ^36^ ^]^ and electron‐beam irradiation.^[^ ^32^, ^33^, ^34^ ^]^ However, these methods often require additional processing steps, degrade the switching medium, and introduce non‐linearity in conductance control.

In this study, we demonstrate reliable analog switching memristors utilizing a few‐layer h‐BN resistive switching medium grown on a two‐inch single‐crystalline gallium nitride (GaN) wafer via MOCVD. The h‐BN films exhibit a unique structure, suspended over GaN nano‐cones, which facilitates the formation of multiple nanoscale contacts between the h‐BN films and the apexes of GaN nano‐cones. This distinctive configuration effectively confines filament formation to the nanoscale contacts. The focused electric field at the nano‐cone apexes reduces the operating voltage, enhancing energy efficiency. Additionally, the gradual switching behavior enabled by multiple nano‐filaments, combined with exceptional in‐plane thermal conductivity of h‐BN, mitigates degradation of the switching medium, ensuring excellent cycle‐to‐cycle consistency. Furthermore, the memristor demonstrates high accuracy in simulations using the Modified National Institute of Standards and Technology (MNIST) database, attributed to its highly linear and symmetric synaptic weight update capability.

## 2 Results and Discussion

**Figure 1:**
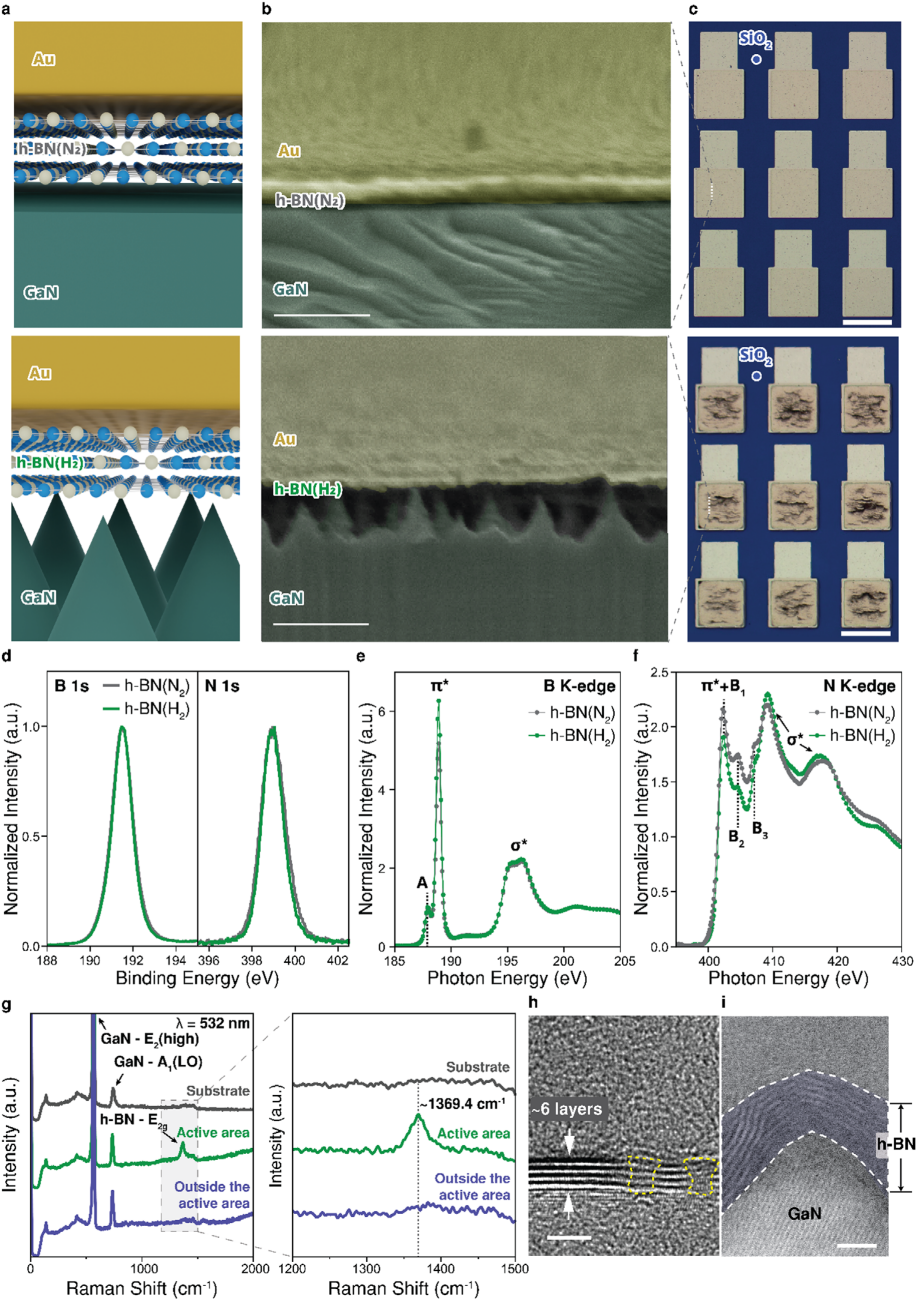
Fabrication and spectroscopic analyses of h‐BN‐based memristors. a) Schematics of an Au/h‐BN(N_2_)/n‐type GaN structured memristor (top) and an Au/h‐BN(H_2_)/n‐type GaN structured memristor (bottom). b) Cross‐sectional SEM images (false‐colored) of an h‐BN(N_2_) memristor (top) and an h‐BN(H_2_) memristor (bottom). The scale bar is 500 nm for both images. c) Optical microscopy images of an h‐BN(N_2_) memristor (top) and an h‐BN(H_2_) memristor (bottom). The device size is 200 × 200 µm^2^ and the scale bar is 200 µm. d) High‐resolution XPS core‐level spectra of h‐BN(N_2_) films (gray) and h‐BN(H_2_) films (green) grown on GaN substrates. Both samples exhibit nearly identical B 1s (left) and N 1s spectra (right). e,f) NEXAFS spectra of e) the B K‐edge and f) the N K‐edge of h‐BN(N_2_) and h‐BN(H_2_) films at the X‐ray incident angle of 55°. g) Raman spectra obtained from a GaN substrate as a reference (gray), the active area (green), and outside the active area (blue) of an h‐BN(H_2_) sample. (λ = 532 nm). h) Cross‐sectional TEM image of six‐layer h‐BN films. The yellow‐colored regions are defective areas in the h‐BN films. The scale bar represents 2 nm. i) False‐color contrast inverted bright‐field STEM of h‐BN(H_2_) films on an apex of a GaN nano‐cone. The scale bar represents 2 nm.

**Figure** **1**a depicts schematic cross‐sectional images of the fabricated memristors with a metal‐insulator‐semiconductor (MIS) configuration, consisting of an Au top electrode and a few layers of h‐BN resistive switching medium grown by MOCVD on an n‐type GaN substrate that serves as the bottom electrode. During the MOCVD growth, triethylborane (TEB) and NH_3_ precursors were delivered to the GaN substrate using two different carrier gases, N_2_ and H_2_. For clarity, h‐BN films grown with N_2_ carrier gas are denoted as h‐BN(N_2_), while those grown with H_2_ are referred to as h‐BN(H_2_). It is important to note that at high temperatures (>1000 °C), the use of H_2_ carrier gas can induce the decomposition of GaN Ga and N, followed by desorption.^[^ ^37^ ^]^ This H_2_‐induced thermal decomposition preferentially occurs at crystalline defects, such as dislocations, within the GaN substrate, ultimately leading to the formation of a unique structure, in which few‐layer h‐BN is suspended over GaN nano‐cones.^[^ ^38^ ^]^

On the other hand, with N_2_ carrier gas, GaN remains chemically stable, allowing h‐BN to grow uniformly on the atomically flat GaN. The distinct morphological differences between Au/h‐BN(N_2_)/GaN and Au/h‐BN(H_2_)/GaN structures are shown in the cross‐sectional scanning electron microscopy (SEM) images in Figure 1b. Optical microscopy images of the fabricated device array are presented in Figure 1c, while the detailed fabrication process is shown in Figure S1 (Supporting Information).

For the comparison of the chemical bonding states of h‐BN films grown under different carrier gases, X‐ray photoelectron spectroscopy (XPS) analysis was employed. Figure 1d presents the XPS spectra of h‐BN(N_2_) and h‐BN(H_2_), both exhibiting B 1s peaks at 191.48 eV and N 1s peaks at 398.96 eV, which are characteristics of sp^2^‐bonded B─N bonds in h‐BN. The full width at half maximum (FWHM) values for the B 1s peaks were 1.16 eV for h‐BN(N_2_) and 1.08 eV for h‐BN(H_2_), while the N 1s peaks exhibited FWHM values of 1.32 and 1.14 eV, respectively. The absence of additional peaks in the XPS core‐level spectra confirms that both samples primarily consist of sp^2^‐hybridized h‐BN without notable chemical impurities or secondary bonding configurations. Although the FWHM values for h‐BN(H_2_) are slightly narrower, indicating marginally improved crystallinity, both samples exhibit sufficiently sharp peaks, suggesting that their overall chemical states and crystalline quality are comparable.

We further analyzed near‐edge X‐ray absorption fine structures (NEXAFS) spectra to investigate the electronic states and bonding configurations of the grown h‐BN layers. Figure 1e,f show NEXAFS spectra of both samples. The peak at 188.9 eV (402.2 eV) and the peak near 195.4 eV (409.2 and 417.6 eV) in the B (N) K‐edge indicate the transition from a B 1s (N 1s) core‐level to a π* or σ* bonding state of sp^2^‐BN films, respectively. We observed a defect‐induced peak at 187.9 eV in the B K‐edge, denoted as A in Figure 1e, which is related to boron‐terminated vacancy defects.^[^ ^39^ ^]^ Notably, the intensities of this peak show no significant difference between the two samples, indicating a similar level of defect concentration. A slight difference was found in three peaks at 402.2, 404.6, and 407.2 eV in the N K‐edge, which are denoted as B_1_, B_2_, and B_3_ in Figure 1f. Three peaks are attributed to the chemical bonding states of GaN substrate below h‐BN films, represent transitions from a N 1s core‐level to π* bonding states (B_1_ and B_3_) and the transition from a N 1s core‐level to a σ* bonding state (B_2_).^[^ ^8^, ^40^ ^]^ It is noteworthy that the three peaks of the h‐BN(H_2_) sample show lower intensity compared to those of the h‐BN(N_2_) sample due to the etched GaN substrate at the topmost layer of the h‐BN(H_2_) sample. The chemical bonding states explored by XPS and NEXAFS confirm that h‐BN films grown under different carrier gases have similar crystallinity with no additional defect‐induced chemical states which may affect the performance of memristors.

We utilized this growth behavior to control the formation of h‐BN/GaN nano‐cones heterostructures within the device's active areas.^[^ ^41^ ^]^ Specifically, a 100 nm SiO_2_ masking layer was applied outside the active area to prevent GaN desorption under H_2_ carrier gas, ensuring that the geometric effects of GaN nano‐cones occur exclusively in the unmasked regions (Figure S2, Supporting Information). As a result, Au/h‐BN(H_2_)/GaN memristors exhibit a rough surface morphology in the active areas due to the h‐BN layer on GaN nano‐cones, whereas Au/h‐BN(N_2_)/GaN memristors maintain a smooth surface consistent with the underlying epitaxial GaN substrate. To quantitatively confirm the topographical changes of Au/h‐BN(H_2_)/GaN memristors induced by GaN nano‐cone formation, atomic force microscopy (AFM) topography measurements were performed before and after the growth of h‐BN(H_2_) films. AFM height mapping images (Figure S3a,b, Supporting Information) clearly illustrate a significant increase in surface roughness following h‐BN(H_2_) growth. The root‐mean‐square (RMS) roughness measured across multiple areas of 1 × 1 µm^2^ increased markedly from 0.495 ± 0.221 nm on the pristine GaN substrate to 9.024 ± 2.566 nm after growth, as shown in Figure S3c (Supporting Information). 3D AFM topography scans over 5 × 5 µm^2^ areas (Figure S4, Supporting Information) further visualize the transition from an atomically flat pre‐growth surface to a uniformly distributed nano‐cone structure after the growth of h‐BN(H_2_) films. The topographical analyses verify the morphological changes induced by H_2_ carrier gas during MOCVD growth, resulting in the rough surface observed for Au/h‐BN(H_2_)/GaN memristors.

Notably, Raman spectra obtained from within or outside the active area exhibit distinct features, confirming the selective growth of h‐BN, as shown in Figure 1g. The active area displays a strong Raman peak at 1369.4 cm^−1^, with a full width at half maximum (FWHM) of 26.1 cm^−1^ (Figure S5, Supporting Information), corresponding to the in‐plane E_2g_ vibration mode,^[^ ^42^ ^]^ whereas no such peak is observed outside the active area. This masking strategy effectively confines h‐BN nucleation and deposition within the unmasked regions, enabling precise patterning of h‐BN/GaN heterostructures, thereby simplifying device design and enhancing operational reliability. Cross‐sectional transmission electron microscopy (TEM) image (Figure 1h) further confirms the presence of h‐BN films, revealing a thickness of ≈2 nm (equivalent to 6–7 atomic layers. Additionally, TEM analysis also reveals the formation of defective regions across the MOCVD‐grown h‐BN films, which serve as conductive paths in memristors, consistent with the observation in the NEXAFS B K‐edge spectra. High‐resolution cross‐sectional scanning transmission electron microscopy (STEM) analysis provides additional structural insights into the structure of h‐BN(H_2_) films. As clearly shown in Figure S6 (Supporting Information), the h‐BN(H_2_) films are suspended over the GaN nano‐cone structures. Figure 1i demonstrates that the apex of a nano‐cone is covered by ≈2 nm‐thick h‐BN films, corresponding to about 6–7 atomic layers. The cross‐sectional TEM images provide clear and direct confirmation of the structural characteristics and morphology of the h‐BN films grown on a GaN substrate.

### 2.1 Geometric Confinement Effects on h‐BN Memristor Performance

**Figure 2:**
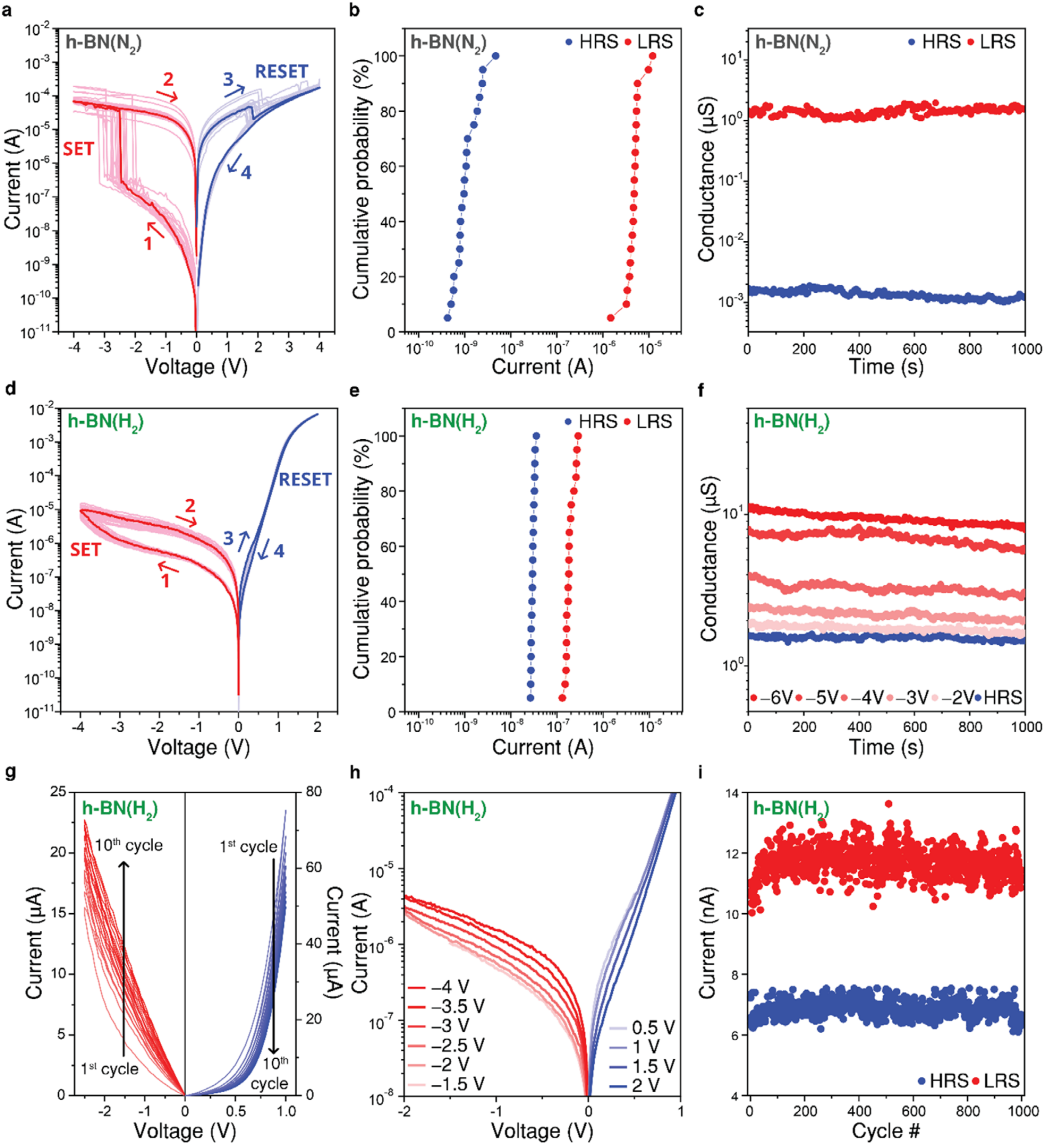
Different switching behaviors and electrical properties of h‐BN memristors with different geometries. a) Digital switching characteristics of the h‐BN(N_2_) memristor without current compliance, measured over 20 repeated DC voltage sweeps. Negative and positive voltage sweeps are applied for the SET and RESET processes, respectively. b) Cumulative probability plots of the current (@ −0.2 V) at the HRS (blue) and LRS (red) measured from the h‐BN(N_2_) memristor. c) Retention properties of the h‐BN(N_2_) memristor at the binary states of HRS (blue) and LRS (red). d) Analog switching characteristics of the h‐BN(H_2_) memristor without current compliance, measured over 20 repeated DC voltage sweeps. Negative and positive voltage sweeps are applied for the SET and RESET processes, respectively. e) Cumulative probability plots of the current (@ −0.2 V) at the HRS (blue) and LRS (red) measured from the h‐BN(H_2_) memristor. f) Multi‐level data retention properties of the h‐BN(H_2_) memristor after DC voltage sweeps of various ranges without current compliance. g) Ten repeated negative DC voltage sweeps (0 V → −2 V → 0 V) for gradual SET and ten repeated positive DC voltage sweeps (0 V → +1 V → 0 V) for gradual RESET of the h‐BN(H_2_) memristor. h) Log‐scale I–V curves of the h‐BN(H_2_) memristor with a series of consecutive voltage sweeps, including 0 V → −1.5 V → 0 V, followed by 0 V → −2 V → 0 V, continuing in steps to −4 V for SET. RESET voltage sweeps include 0 V → +0.5 V → 0 V, then 0 V → +1 V → 0 V, continuing in steps to +2 V for RESET. Voltages of −1.5 V and +0.5 V are sufficient to enable SET and RESET, respectively. i) Endurance test of the h‐BN(H_2_) memristor with repeated cycles of SET (−2 V for 1 s) and RESET (+1.3 V for 1 s) pulses. A read pulse (−0.2 V for 1 s) is applied after each pulse to measure the updated conductance.

Resistive switching behaviors of Au/h‐BN(N_2_)/GaN and Au/h‐BN(H_2_)/GaN memristors are investigated to confirm the geometric confinement effects on the performance of memristors. Digital switching characteristics, characterized by abrupt SET and RESET during DC voltage sweeps, were observed for the Au/h‐BN(N_2_)/GaN memristor (h‐BN(N_2_) memristor), as shown in **Figure** **2**a. Notably, the h‐BN(N_2_) memristor required an electroforming process involving a relatively high voltage and a compliance current, as illustrated in Figure S7 (Supporting Information). We performed more than 20 cycles of SET and RESET procedures, which resulted in stochastic switching with average SET and RESET voltages of −2.62 and 1.98 V, respectively. Current levels at HRS and LRS varied since the size of the CFs could not be precisely controlled without the current compliance setting.^[^ ^43^ ^]^ Therefore, the cumulative probability of the h‐BN(N_2_) memristor in Figure 2b shows high cycle‐to‐cycle variations, with average current levels of 1.33 nA at HRS and 5.06 µA at LRS. The device exhibits good long‐term data retention for more than 10^3^ s at the binary states of HRS and LRS (Figure 2c).

Notably, the Au/h‐BN(H_2_)/GaN memristor (h‐BN(H_2_) memristor) showed quite different I‐V characteristics, that is, gradual increase and decrease in SET and RESET, respectively, as shown in Figure 2d. This suggests that nanoscale contacts between h‐BN films and the apexes of GaN nano‐cones play a pivotal role in gradual, rather than sudden, switching behavior for h‐BN(H_2_) memristors. Interestingly, h‐BN(H_2_) memristors exhibited an electroforming‐like behavior distinct from conventional electroforming processes, which typically involve applying high voltages with a compliance current. As demonstrated by initial sweeps in Figure S8 (Supporting Information), the memristors showed an increase in the current level of the HRS following the first sweep, indicating a mild, compliance‐free electroforming process. The distributions of current levels of h‐BN(H_2_) memristors were highly uniform, indicating low cycle‐to‐cycle variability with average current levels of 29.8 nA at HRS and 193 nA at LRS as shown in Figure 2e. Stable switching was achieved with consecutive cycles, as gradual switching of h‐BN(H_2_) memristors reduces degradation of h‐BN films. Statistical analyses across 10 devices show that h‐BN(H_2_) memristors exhibit reduced variability in both HRS and LRS compared to h‐BN(N_2_) devices, consistent with the cycle‐to‐cycle variation. Current distributions and variability metrics are presented in Figures S9–S11 (Supporting Information). Figure 2f shows the retention property after negative DC voltage sweeps of various ranges without current compliance setting. Note that the h‐BN(H_2_) memristor demonstrates excellent long‐term retention of multi‐level states, maintaining stability for over 10^3^ s. As observed in Figure 2c, while the h‐BN(N_2_) memristor exhibits binary states, the h‐BN(H_2_) memristor enables multi‐level operation, which is a key requirement for analog switching. Retention tests at various temperatures confirm the stability of h‐BN(H_2_) memristors, as shown in Figure S12 (Supporting Information). At 100 K, conductance remains stable for 1000 s, whereas at 260 K, stability persists with minor fluctuations.

To clarify the conductance below G_0_ observed in h‐BN memristors, we highlight the structural difference from conventional metal‐insulator‐metal configurations, as the devices are based on MIS structures. MIS structures inherently include interface scattering, limited carrier mobility, and additional series resistance from the semiconductor bottom electrode, preventing ballistic transport and thus leading to conductance below the ideal G_0_.^[^ ^44^ ^]^ We measured the I‐V characteristics of an Au/GaN/Au structure, as exhibited in Figure S13 (Supporting Information), which revealed a significant series resistance near 2.75 kΩ, confirming substantial voltage drops across the GaN bottom electrode.

To exhibit the gradual switching of the h‐BN(H_2_) memristor, 10 repeated negative DC voltage sweeps were carried out with voltage sequence of 0 V → −2 V → 0 V for SET and then 10 repeated positive DC voltage sweeps 0 V → 1 V → 0 V for RESET (Figure 2g). We observed incremental and decremental current levels under repeated negative and positive voltage sweeps, respectively, indicating that multi‐level states of the h‐BN(H₂) memristor with analog switching behavior can be achieved. Figure 2h exhibits repeated DC voltage sweeps with incremental range to confirm SET and RESET voltages of the h‐BN(H_2_) memristor. The SET voltage of −1.5 V was enough to increase the conductance of the devices, which was lower than the SET voltage of the h‐BN(N_2_) memristor. Likewise, the RESET voltage of 0.5 V was also lower than that of the h‐BN(N_2_) memristor. The h‐BN(H_2_) memristor with nano‐cone structures operates at a lower voltage and current level, making it more energy‐efficient.^[^ ^45^, ^46^ ^]^ Its reduced operation voltage further highlights its advantage in lowering power consumption. Figure 2i shows high endurance of the h‐BN(H_2_) memristor with consistent current levels at the LRS and HRS >1000 cycles.

To investigate the switching mechanisms of both h‐BN memristors, temperature‐dependent I‐V measurements were carried out on the h‐BN(N_2_) and h‐BN(H_2_) memristors over the 140–260 K temperature range.^[^ ^47^ ^]^ Arrhenius plots in both devices reveal transitions in conduction from the high‐resistance state to the low‐resistance state, as shown in Figure S14 (Supporting Information).

For the h‐BN(N_2_) memristor, the activation energy (E_a_) decreases with increasing voltage in the HRS, confirming thermally activated conduction, as illustrated in Figure S15a (Supporting Information). After the SET process, E_a_ remains ≈3 meV regardless of the applied voltage, a clear indicator of vacancy‐based filament formation.^[^ ^48^, ^49^ ^]^ Double‐logarithmic I–V analysis reveals a slope of 1 in the LRS, as illustrated in Figure S15b (Supporting Information), consistent with Ohmic behavior and the presence of a vacancy‐based filament, as presented in Figure S16 (Supporting Information).

h‐BN(H_2_) memristors also exhibit positive E_a_ in the HRS that decreases with increasing voltage, suggesting thermally activated conduction, as shown in Figure S17a (Supporting Information). After SET, the LRS exhibits negative E_a_, as shown in Figure S17b (Supporting Information). Negative E_a_ has been reported in memristive systems where conduction occurs via formed metallic filaments,^[^ ^47^, ^50^, ^51^ ^]^ which typically exhibit near zero or negative activation energies due to a temperature‐dependent resistance increase from electron‐phonon scattering. In the low‐bias region of the LRS in the h‐BN(H_2_) memristor, the observed negative E_a_ originates from conduction through conductive paths formed within h‐BN films, in which increased lattice vibrations reduce tunneling probability as temperature rises. In contrast, at high bias, a strong electric field lowers defect barriers and enables field‐enhanced tunneling, thereby diminishing the effect of temperature on conduction and reducing the absolute value of negative E_a_.

Given that the LRS of the h‐BN(H_2_) memristor exhibits negative activation energy, thermally activated conduction mechanisms can be excluded. Therefore, we focused our analysis on non‐thermally activated conduction mechanisms such as direct tunneling and Fowler‐Nordheim tunneling, employing field‐dependent fittings including double‐logarithmic current‐voltage, ln I versus 1/E and ln(I/V^2^) versus 1/V.

Double‐logarithmic current‐voltage analysis shown in Figure S18a (Supporting Information) demonstrates Ohmic conduction through nano‐filaments at lower bias and a shift to a higher slope under higher bias conditions, indicating weakly formed filaments within h‐BN films.^[^ ^52^ ^]^ Similar behavior has been observed in metal‐oxide‐based VCM with variable oxygen vacancy concentration.^[^ ^53^ ^]^ ln(I/V^2^)–1/V plots eliminate any linear Fowler‐Nordheim regions and highlight nonlinear defect‐mediated tunneling processes, as shown in Figure S18b (Supporting Information). ln I versus 1/E plots of Figure S18c (Supporting Information) show curvature that corresponds to local modulation of defect barriers under higher fields.

These findings demonstrate that h‐BN(N_2_) memristors rely on stable filaments, while h‐BN(H_2_) memristors form defect‐mediated nano‐filaments. The filament formation in both memristors is potentially attributed to the migration of B vacancies since the SET process occurs at negative bias and the bottom electrode of GaN is an electrochemically inert material.^[^ ^54^, ^55^ ^]^

### 2.2 Observation of Multiple Confined Nano‐Filaments

**Figure 3:**
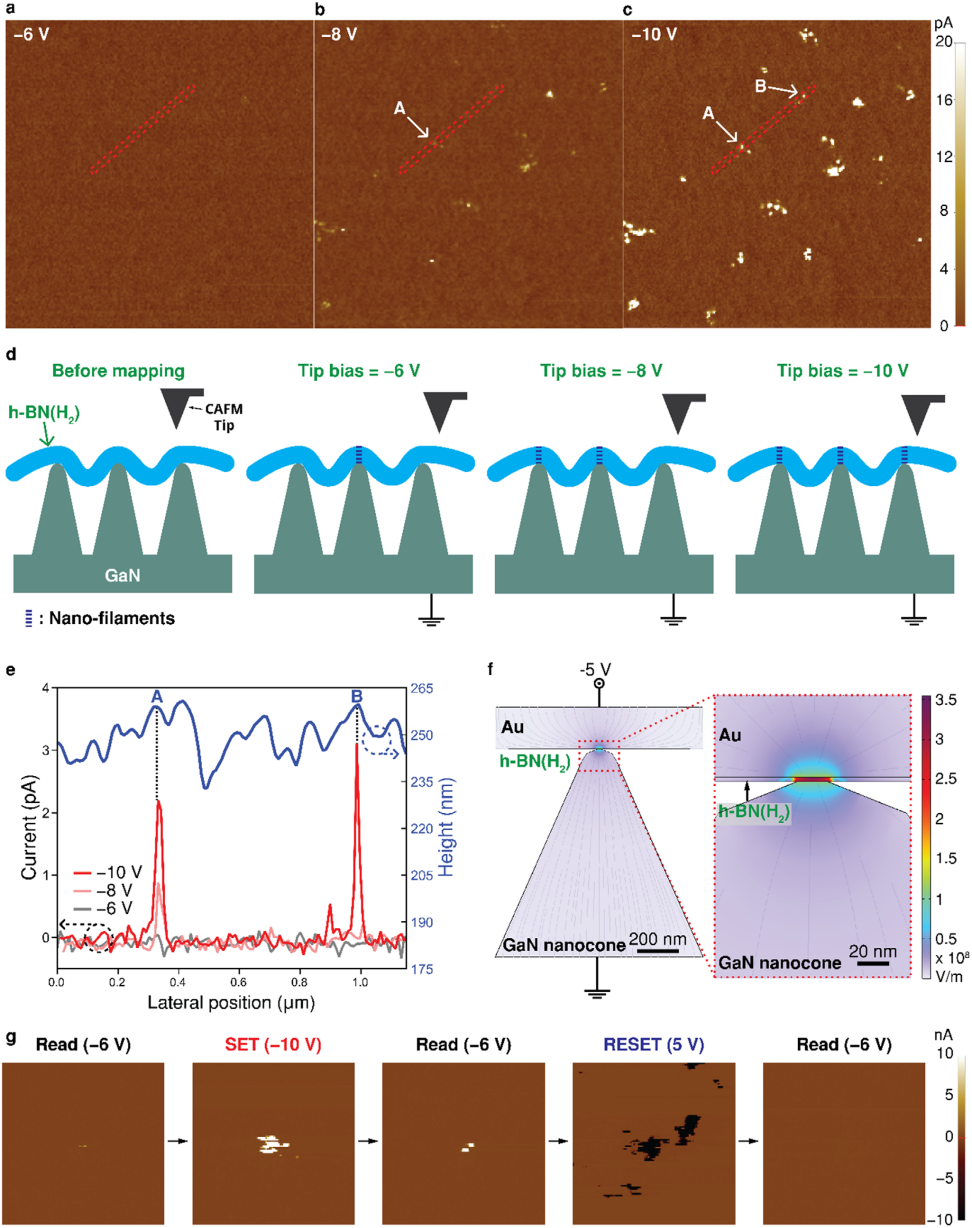
Observation of multiple nano‐filament confinements. a–c) Consecutive CAFM current mapping images measured at the same area of 2.5 × 2.5 µm^2^ under incremental tip biases of a) −6 V, b) −8 V, and c) −10 V applied on the h‐BN(H_2_) surface. The color bar indicates a current range from 0 to 20 pA. d) Schematics of the gradual formation of multiple nano‐filaments while CAFM current mapping under increasing tip bias e) Height and current line profiles at the red squared region in a–c). f) Electrical potential distributions of an Au/h‐BN(H_2_)/GaN nano‐cone structured memristor during the SET process simulated using COMSOL Multiphysics 6.0. g) Sequence of CAFM current maps obtained from the same 1 × 1 µm^2^ area on h‐BN(H_2_) films. The sequence includes: (1) initial read at −6 V, (2) SET process at −10 V, (3) post‐SET read at −6 V, (4) RESET process at 5 V, and (5) post‐RESET read at −6 V. A localized conduction path appears after the SET process and is erased after RESET, confirming the reversible formation and rupture of the filament. The color bar indicates a current range from −10 to 10 nA.

In order to observe the formation of conduction paths between h‐BN(H_2_) and GaN nano‐cones, we performed conductive atomic force microscopy (CAFM) current mapping analysis of the h‐BN(H_2_) films suspended over GaN nano‐cones under various voltage conditions. The current mapping images in **Figure** **3**a–c were consecutively captured under applied voltages of −6, −8, and −10 V, respectively, over the same 2.5 × 2.5 µm^2^ area. No current flow through h‐BN films was observed at the applied voltage of −6 V. However, confined current paths appeared at −8 V, and the number of paths increased at −10 V. Notably, as the applied voltage increased, new current paths formed at additional contact points between the h‐BN films and GaN nano‐cones. As shown in Figure 3d, increasing the tip bias to mimic the negative DC voltage sweep induces multiple nano‐filaments formation, resulting in the gradual switching behavior of the h‐BN(H_2_) memristor. Figure 3e shows a combined plot of the current and morphology line profiles for the suspended h‐BN films, obtained from the exact same position within the red dotted area in Figure 3a–c. When the voltage increased to −10 V, a new current path appeared at position B without the increase in the diameter of the existing current path formed at −8 V. The results indicate that conduction predominantly occurs at apexes of GaN nano‐cones, where enhanced electric fields promote defect formation within confined regions of the h‐BN films, enabling the gradual conductance modulation, i.e., analog h‐BN(H_2_) memristors. The observed conduction behavior provides strong evidence that the filament‐based mechanism in h‐BN(H_2_) memristors is highly localized. To emulate similar situations of synaptic weight updates, we repeated current mapping at the same area under constant bias. We observed increasing numbers of nano‐filaments after repeated current mapping (Figure S19, Supporting Information). The gradual formation of multiple nano‐filaments under constant electrical stimuli can enable linear synaptic weight updates, as will be demonstrated in the following section.

Electric potential distributions near the interface between h‐BN and the apex of a GaN nano‐cone are simulated with COMSOL Multiphysics software. As shown in Figure 3f, the electric field is focused near the apex of GaN nano‐cones, facilitating the formation of confined nano‐scale filament at relatively low applied voltage. We confirmed that the focused electric field of 3.18 × 10^8^ V m^−1^ at the nanoscale contact point is about 45.6 times higher than the electric field in suspended region (6.97 × 10^6^ V m^−1^), leading to the predominant formation of filaments confined at the apex of GaN nano‐cones. Furthermore, Figure S20 (Supporting Information) shows that the electric field in the h‐BN films on flat GaN is relatively lower at 1.86 × 10^7^ V m^−1^ compared to that in the h‐BN(H_2_), indicating a much higher filament formation threshold voltage, consistent with the result of a higher operating voltage of h‐BN(N_2_) memristors.

To further verify the non‐volatility and reversibility of filamentary switching, we conducted sequential CAFM current mapping on the same area of 1 × 1 µm^2^ of h‐BN(H_2_) films. As shown in Figure 3g, a series of five measurements were performed in the following order of initial read at −6 V, SET at −10 V, post‐SET read at −6 V, RESET at 5 V, and post‐RESET read at −6 V. A localized current spot emerges after the SET process, confirming the formation of a non‐volatile conductive filament. Following the RESET operation, this signal completely disappears, indicating full rupture of the previously formed filament. These observations provide direct evidence of the non‐volatile formation and reversible rupture of filaments under electrical stimulus.

### 2.3 Linear Synaptic Weight Updates via Multiple Nano‐Filaments Confinement

**Figure 4:**
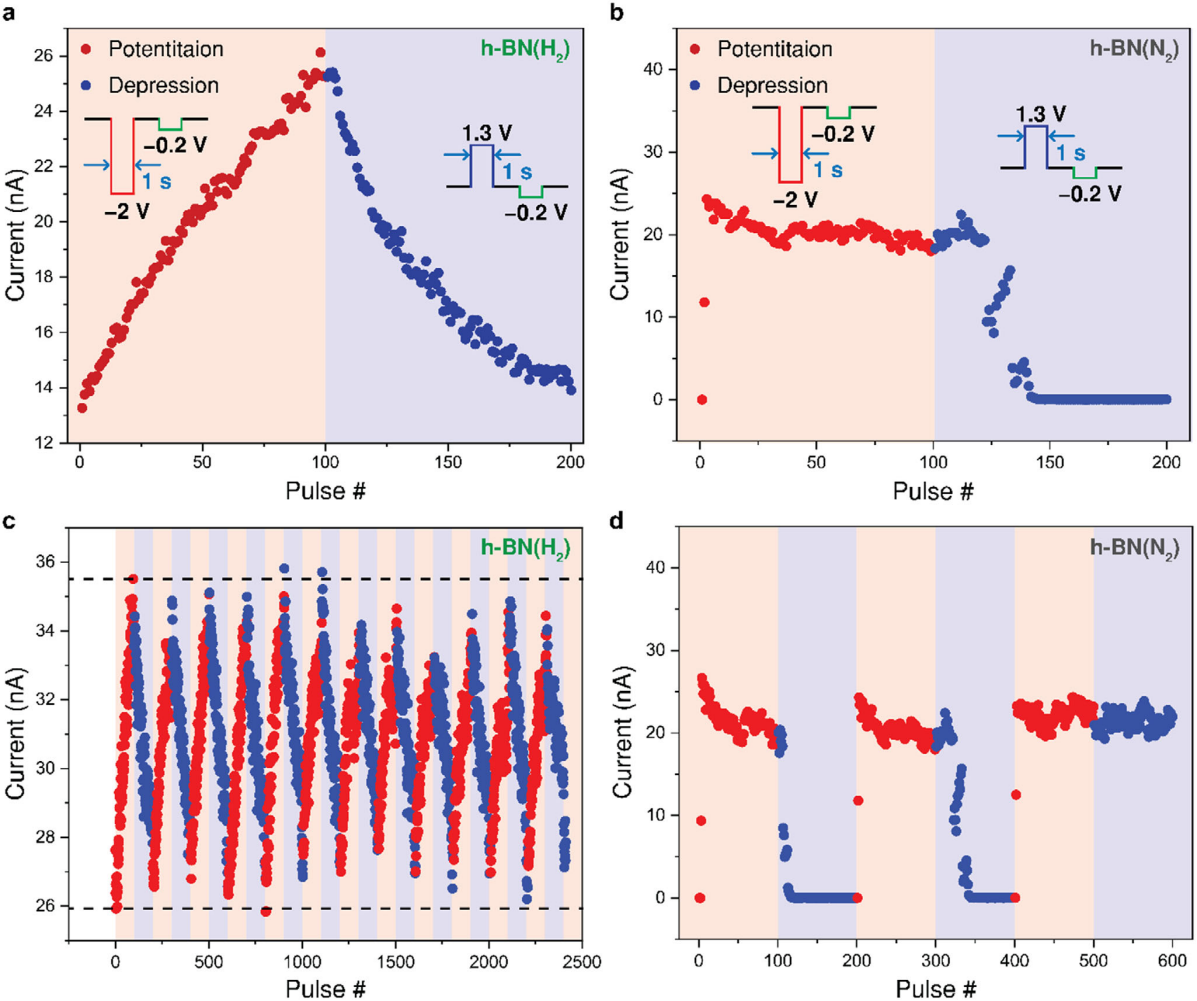
Synaptic plasticity in h‐BN‐based memristors exhibiting analog and abrupt switching behaviors. a) LTP and LTD characteristics of a h‐BN(H_2_) memristor. 100 potentiation pulses (−2 V, 1 s) and 100 depression pulses (1.3 V, 1 s) are applied for synaptic weight updates. A read pulse (−0.2 V, 1 s) is applied after each pulse to monitor conductance changes. b) LTP and LTD characteristics of a h‐BN(N_2_) memristor under identical conditions as a). c) 12 repeated cycles of 100 potentiation and 100 depression pulses in a h‐BN(H_2_) memristor. d) 3 consecutive cycles of 100 potentiation and 100 depression pulses in an h‐BN(N_2_) memristor, exhibiting irreversible SET in the third cycle.

We performed consecutive 100 potentiation and 100 depression pulses to confirm the long‐term potentiation (LTP) and depression (LTD) behavior of h‐BN(H_2_) and h‐BN(N_2_) memristors for analog deep learning accelerators. For LTP, a −2 V pulse with a 1‐second duration was applied, and for LTD, a 1.3 V pulse with a 1‐second duration was used. To read the updated synaptic weights after each update, a read pulse of −0.2 V with a 1‐second duration was also applied for both devices. Interestingly, LTP and LTD in the h‐BN(H_2_) memristor, as shown in **Figure** **4**a, exhibit linear and symmetric characteristics, whereas those in the h‐BN(N_2_) memristor, shown in Figure 4b, display nonlinear and asymmetric characteristics due to abrupt transitions. To assess long‐term stability of linear synaptic weight updates of the h‐BN(H_2_) memristor, retention characteristics were measured after applying various potentiation pulses, as shown in Figure S21 (Supporting Information). The ability to preserve multiple conductance states over extended periods confirms that the memristors exhibit long‐term synaptic plasticity. The LTP and LTD characteristics could be modeled based on the following equation:^[^ ^56^ ^]^

(1) $$\mathrm{Potentiation}:G_{P}\left(n\right)=\frac{\left(G_{max}-G_{min}\right)}{\left(1-e^{-\alpha_{P}\times P_{max}}\right)}\times \left(1-e^{-\alpha_{P}\times n}\right)+G_{min}$$

(2) $$\begin{array}{ccc} \mathrm{Depression}:G_{D}\left(n\right) & = & -\frac{\left(G_{max}-G_{min}\right)}{\left(1-e^{-\alpha_{D}\times P_{max}}\right)}\times \left(1-e^{-\alpha_{D}\times \left(P_{max}-n\right)}\right)\\ & & +G_{max}, \end{array}$$

where *G_P_* and *G_D_* refer to the updated conductance values after potentiation and depression, respectively. *G_max_* and *G_min_* represent the measured maximum and minimum conductance values, respectively. *P_max_* denotes the number of pulses applied for updates, and n indicates the specific pulse number. Based on the model, for the h‐BN(H_2_) memristor, fitting the measured data shows alpha values of 0.00487 for potentiation and 0.02012 for depression (non‐linearity factors of ν^+^ = 0.487, ν^−^ = 2.012). In contrast, for the h‐BN(N_2_) memristor, fitting the measured data exhibits alpha values of 0.39681 for potentiation and 0.02919 for depression (non‐linearity factors of ν^+^ = 39.68, ν^−^ = 2.918). These results indicate that the nano‐filaments confinement between the GaN nano‐cones and the h‐BN(H_2_) films enables linear synaptic weight updates. Figure 4c,d presents consecutive weight updates for both samples, demonstrating that the abrupt switching behavior observed in the h‐BN(N_2_) memristor hinders the repeatability required for reliable synaptic weight updates.

To evaluate the accuracy of the h‐BN(H_2_) memristor for ANNs training, we simulated an ANN with the MNIST handwritten classification tasks using the Tiki‐Taka algorithm by utilizing the IBM aihwkit framework.^[^ ^56^ ^]^ Slight asymmetry might cause learning failures, but this could be overcome using the Tiki‐Taka algorithm, which mitigates the asymmetric update characteristic.^[^ ^57^ ^]^ As shown in Figure S22a (Supporting Information), the artificial neural network was organized with 784 pre‐neurons, 256 neurons for the first hidden layer, 128 neurons for the second hidden layer, and 10 output neurons for representing input digits from 0 to 9. Each MNIST handwritten digit image was divided into 28 × 28 pixels, corresponding to the 784 pre‐neurons. To account for the representativeness of the simulation and the variability of the h‐BN memristors, a 10% variation was introduced as a non‐ideal factor in both models. As shown in Figure S22b (Supporting Information), the h‐BN(H_2_) memristor achieved an accuracy of 96.2%, which is comparable to the floating‐point (FP) baseline accuracy of 98.1% after 30 training epochs. In contrast, the h‐BN(N_2_) memristor exhibited a relatively lower accuracy of 93.9%. These results suggest that the analog switching behavior enabled by multiple nano‐filament confinement in the h‐BN(H_2_) memristor is more suitable for artificial neural network training than the abrupt switching behavior of the h‐BN(N_2_) memristor.

## 3 Conclusion

Abrupt switching behavior in memristors poses significant challenges for realizing memristor‐based ANNs due to its adverse effects on variation, linearity, and symmetry. We have successfully fabricated MIS‐structured memristors, featuring MOCVD‐grown h‐BN films suspended over GaN nano‐cones with Au top electrodes, to achieve analog switching without the need for a current compliance unit such as transistors. Our unique structure enhances memristor performance through three key mechanisms: (i) the confinement of multiple nano‐filaments, which enables multi‐level analog switching by geometrically limiting the diameter of conductive paths, (ii) reduced degradation from Joule heating due to nano‐scale filament formation in h‐BN films, leading to uniform cycle‐to‐cycle variation and (iii) a highly focused electric field at the apexes of GaN nano‐cones, which reduces operating voltage and lowers energy consumption. The formation of multiple nano‐filaments in h‐BN(H_2_) memristors was further confirmed via CAFM under various bias conditions, providing direct evidence for the observed analog switching behavior. Moreover, the analog switching characteristics achieved by our memristors enable high linear and symmetric LTP and LTD. In MNIST‐based handwritten digit classification tasks, the h‐BN memristor with analog switching behavior achieves a classification accuracy of 96.2%, approaching the digital accuracy of 98.1%. This study provides a novel solution to overcoming the fundamental challenges of conventional memristors and 1T1M configuration for memristor‐based ANNs, paving the way for advancements in next‐generation neuromorphic computing systems.

## 4 Experimental Section

### MOCVD Growth of h‐BN Films on SiO_2_ Patterned GaN Wafer

An n‐type GaN substrate as a bottom electrode was grown on a c‐plane sapphire substrate using a multi‐wafer (11 × 2‐inch) MOCVD system (AIXTRON G3). Trimethylgallium (TMGa) and ammonia (NH_3_) were injected as precursors for gallium and nitrogen, respectively. First, a GaN buffer layer is grown at 550 °C and 400 mbar with NH_3_ and TMGa flow rates of 5000 and 30 sccm, respectively. A first high‐temperature undoped GaN (u‐GaN) layer was deposited at 1040 °C and 400 mbar for 5 min using NH_3_ and TMGa flow rates of 5000 and 60 sccm, respectively. Subsequently, a second u‐GaN layer was grown at 1050 °C and 400 mbar for 5 min with NH_3_ and TMGa flow rates up to 8000 and 100 sccm, respectively. The growth conditions were maintained after stabilizing for 15 min. An n‐type doped GaN layer is grown at 1050 °C using NH_3_ and TMGa flow rates of 8000 and 100 sccm, respectively. A 100 nm SiO_2_ layer was deposited before the growth utilizing plasma‐enhanced chemical vapor deposition (PECVD) instrument (HiDep‐SC, BMR Technology). The SiO_2_ layer prevents the underlying GaN from being affected by carrier gases, as a SiO_2_ layer is typically utilized as a masking layer for MOCVD growth.^[^ ^58^, ^59^ ^]^ To define the pattern of active area, photolithography is implemented. The SiO_2_ layer is patterned into a 200 × 200 µm^2^ active area for each device using wet etching with a dilute hydrofluoric acid (HF) solution. h‐BN films were grown on SiO_2_ patterned epitaxial GaN wafers utilizing the same MOCVD system with different carrier gases of N_2_ and H_2_. Triethylborane (TEB) and ammonia (NH_3_) were injected as precursors for boron and nitrogen, respectively. A pulsed source‐injection mode was utilized to mitigate undesired parasitic reactions between gas‐phase sources. Each pulse cycle consisted of four steps: (1) TEB injection for 5 s, (2) interruption for 2 s, (3) NH_3_ injection for 4 s, and (4) interruption for 2 s. The entire growth consisted of 100 pulse cycles. h‐BN films were grown at 1050 °C and 600 mbar using NH_3_ and TEB flow rates of 8000 and 20 sccm, respectively. N_2_ or H_2_ carrier gas was continuously flowed into the MOCVD reactor while maintaining the total gas flow constant during the h‐BN growth.

### Device Fabrication

After the growth of h‐BN films, 200 nm Au top electrodes are patterned utilizing photolithography and an electron beam evaporator with a deposition rate of 0.1 Å s^−1^ on the h‐BN films, forming an active area of 200 × 200 µm^2^. Au is chosen as the top electrode due to its electrochemical inertness, which makes it resistant to migration under a strong electrical field. A GaN bottom electrode is connected to a gold pad for contact. The fabrication process of Au/h‐BN/GaN memristors is presented in Figure S1 (Supporting Information).

### Synchrotron X‐Ray Radiation Analysis

High‐resolution XPS and NEXAFS spectra were obtained using synchrotron X‐ray radiation from the 10D and 4D beamlines at the Pohang Accelerator Laboratory (PAL), Republic of Korea, to compare the chemical bonding states of h‐BN(N_2_) and h‐BN(H_2_) films. The B 1s and N 1s core‐level spectra of h‐BN films were measured using incident X‐ray beam energies of 250 and 500 eV, respectively. The NEXAFS spectra were acquired using a tilted incident X‐ray angle of 45° and total electron yield (TEY) detection mode.

### Structural Characterization

The morphologies of h‐BN(N_2_) and h‐BN(H_2_) films grown on a GaN substrate were observed with a field‐emission scanning electron microscope (PHILIPS, SXS30s FEG) under an acceleration voltage of 5 kV. To measure the thickness of h‐BN(H_2_) films, cross‐sectional high‐resolution scanning transmission electron microscopy (HR‐STEM) analysis is conducted under an accelerating voltage of 80 kV using a STEM (ARM‐200F, JEOL Ltd., Japan) equipment with a probe corrector (ASCOR, CEOS GmbH, Germany) at Materials Imaging & Analysis Center of POSTECH.

### Electrical Characterization

Electrical characterizations were conducted in ambient air. Prior to testing, all probes are connected to the device electrodes. RVS (Ramped voltage stress) measurements of h‐BN‐based memristors are performed using a Keithley 4200 Source Measurement Unit (SMU). A compliance current of 100 µA was applied only for h‐BN(N_2_) memristor measurements to avoid permanent breakdown due to abrupt switching behavior of the device. For PVS (Pulsed voltage stress) measurements, both probes were connected to two Keithley 2636B SMU. To achieve precise control and efficient data acquisition, the experimental sequence was managed by an in‐house‐developed Python suite. During pulse application, the bottom electrode was grounded via an SMU, while voltages of −2.0 V for potentiation and +1.5 V for depression were applied to the top electrode to modulate device conductance. Additionally, a reading voltage of −0.2 V is applied to the top electrode. The use of asymmetric pulse voltages during potentiation and depression was optimized to achieve improved modulation behavior. Temperature‐dependent I–V measurements were measured by using a chamber probe station equipped with a Keithley 4200 SMU. The device temperature was externally controlled in the range of 140∼260 K using liquid nitrogen and a heating stage. Voltage sweeps for temperature‐dependent I‐V measurements were conducted from 0 to −1 V to mitigate any filament rupture caused by positive bias. Low‐resistance states in both h‐BN(H_2_) and h‐BN(N_2_) memristors were formed after repeated −6 V sweeps to ensure stable LRS.

### Conductive Atomic Force Microscopy (CAFM) Measurement

Both topographic and current mapping images of h‐BN films on GaN nano‐cones were measured under various biases to the substrate using contact mode AFM (NX7, Park systems) with CAFM Cantilever (CONTSCPt 10U). A scan rate of 1 Hz and a maximum gain of 10 nA were applied by using the external CAFM amplifier for the measurement.

### COMSOL Multiphysics Simulation

Three physical modules of Heat transfer in Solid, Electric current, and Electrostatics were conducted for the simulations. All external boundaries of the structure were electrically insulated. The voltage bias was applied at the top electrode, and the bottom electrode was grounded. Material parameters that were used for this modeling are shown in **Table** **1**.

**Table 1:** Material parameters for COMSOL simulations.

Material	Electrical conductivity [S m^−1^]	Density [kg m^−3^]	Relative permittivity	Thermal conductivity [W m^−1^ K^−1^]
Au	45.6 × 10^6^	19320	6.9	315
h‐BN	1 × 10^−8^	2100	3	751
GaN	1.6 × 10^−4^	6150	8.9	220

### MNIST Simulations Utilizing the Tiki‐Taka Algorithm

The neural network training performance was evaluated using a 3 fully connected‐layer model (784‐256‐128‐10) and Modified National Institute of Standards and Technology (MNIST) dataset by utilizing IBM's aihwkit framework.^[^ ^56^ ^]^ Instead of using a stochastic gradient descent algorithm, the Tiki‐Taka algorithm was implemented, which enabled a mechanism similar to mini‐batch gradient descent via two crossbar arrays in an analog cross‐point array.^[^ ^57^, ^60^ ^]^ The forward and backward passes were performed using the Core (C) array, which represents the actual weights, while the immediate weight updates occur in the auxiliary (A) array. This auxiliary array was referred to update the Core array sparsely. The learning rate from C to A was set to 0.05, while the transfer learning rate from A to C was set to 0.01. Based on the measured update characteristics of the device, the weights were mapped accordingly, and the reference conductance was set at the symmetry point of the modeled characteristic.^[^ ^61^ ^]^ When the device‐to‐device variation of all characteristics was assumed to be 10%, the accuracy reaches 96.2% for the h‐BN(H_2_) memristor and 93.9% for the h‐BN(N_2_) memristor.

## Conflict of Interest

The authors declare no conflict of interest.

## Supporting information



Supporting Information

## Data Availability

The data that support the findings of this study are available from the corresponding author upon reasonable request.
