# Supplementary material for: Analog Switching in Hexagonal Boron Nitride Memristors via Multiple Nano‐Filaments Confinement
Source: Small. 2025 Jun 16;21(32):2504507. doi: 10.1002/smll.202504507 (PMC12366253; doi:10.1002/smll.202504507)
Supplement: Supplementary file 1 — Supporting Information [file SMLL-21-2504507-s001.docx]

Supporting Information

Analog Switching in Hexagonal Boron Nitride Memristors via Multiple Nano-Filaments Confinement

Jaesub Song, Seokho Moon, Jinho Byun, Jiye Kim, Junyoung Choi, Hyunjeong Kwak, Inyong Hwang, Changwook Ji, Seyoung Kim and Jong Kyu Kim^*^


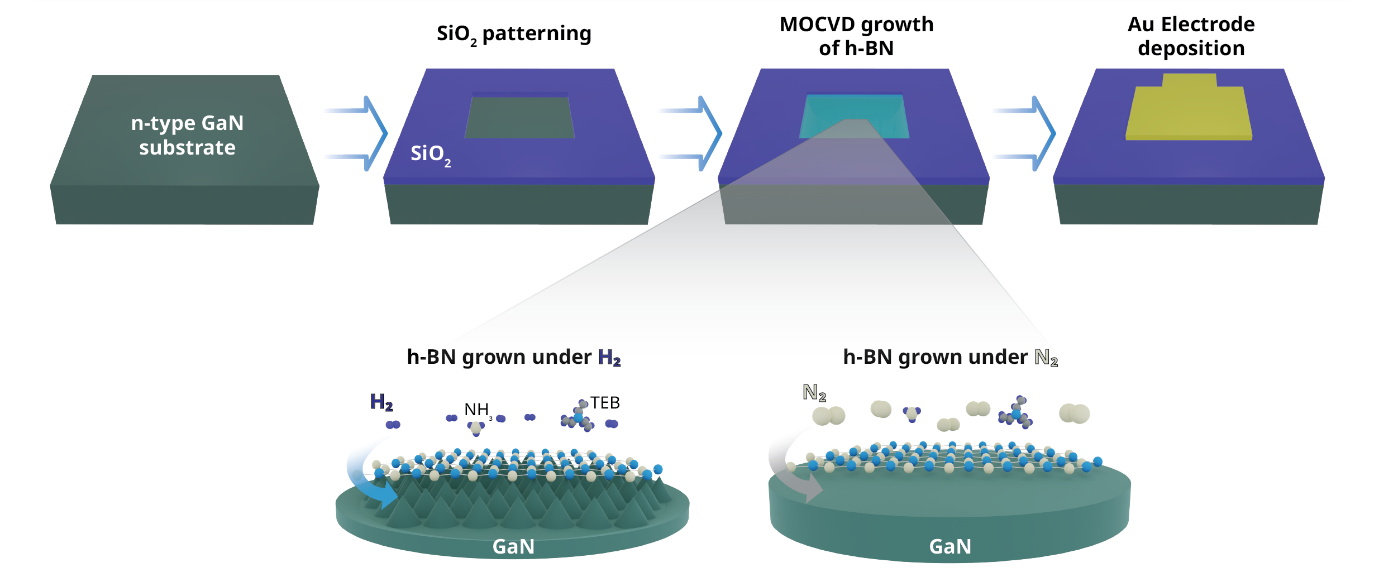


**Figure S1.** The fabrication process of h-BN based memristors.


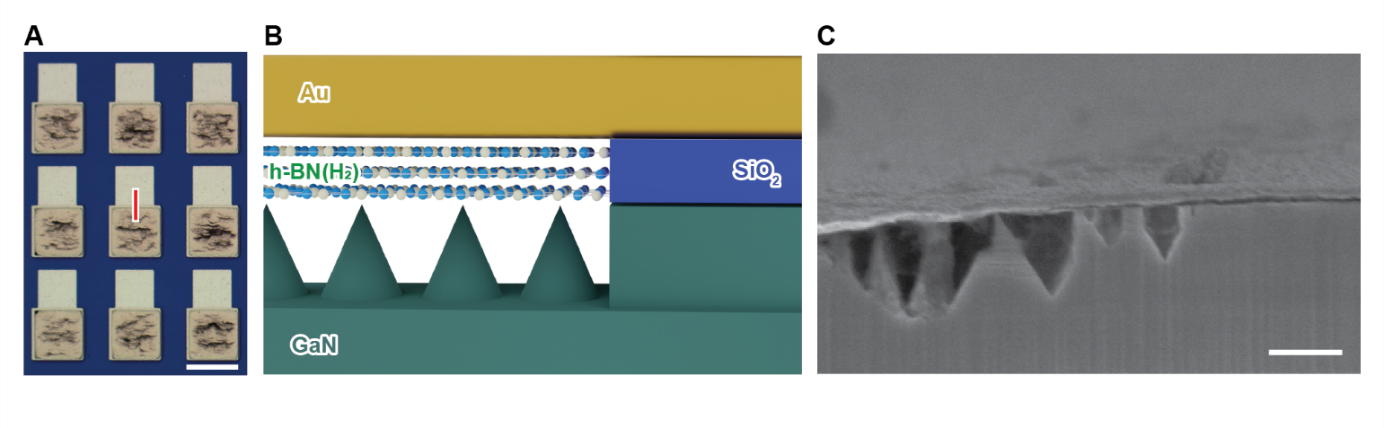


**Figure S2.** Device structure of h-BN(H_2_) memristors. (a) Optical microscopic image of h-BN(H_2_) memristors. The scale bar represents 200 μm. (b) Schematic image of cross-sectional structure in the red-lined region in (a). (c) Cross-sectional SEM image of the red-lined region in (a). The scale bar represents 500 nm.


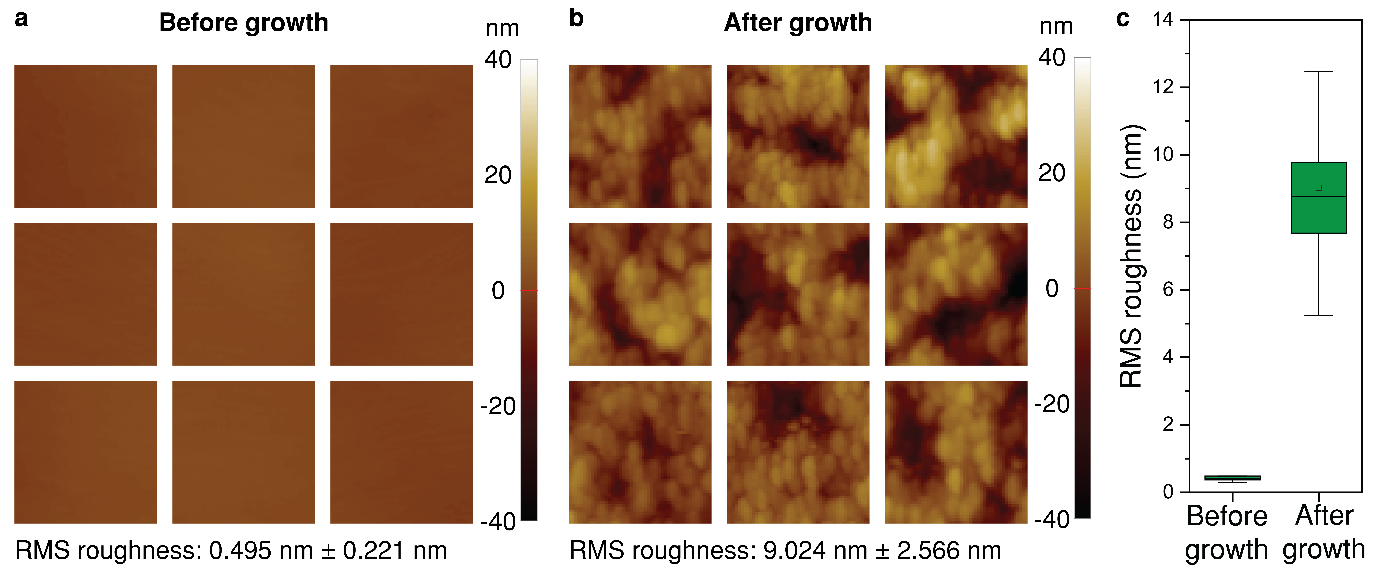


**Figure S3.** AFM height mapping images of nine 1 × 1 μm^2^ regions on (a) the GaN substrate before growth and (b) the surface after MOCVD growth of h-BN(H_2_) films. (c) Box plot comparing the RMS roughness values before and after growth.


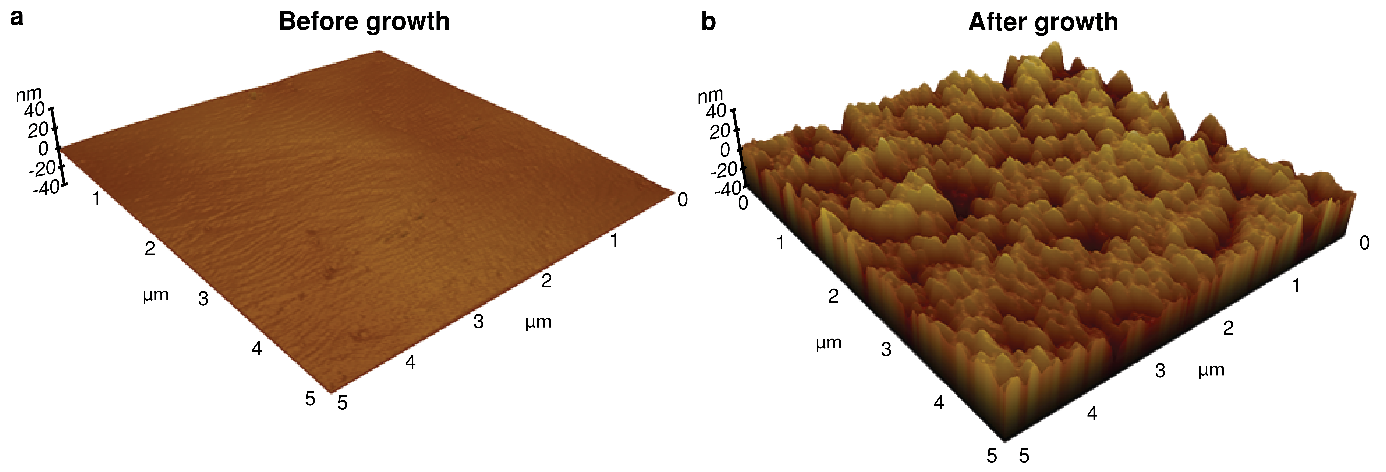


**Figure S4.** Three-dimensional AFM topography images acquired over 5 × 5 μm^2^ areas (a) before and (b) after the growth of h-BN(H_2_) films.


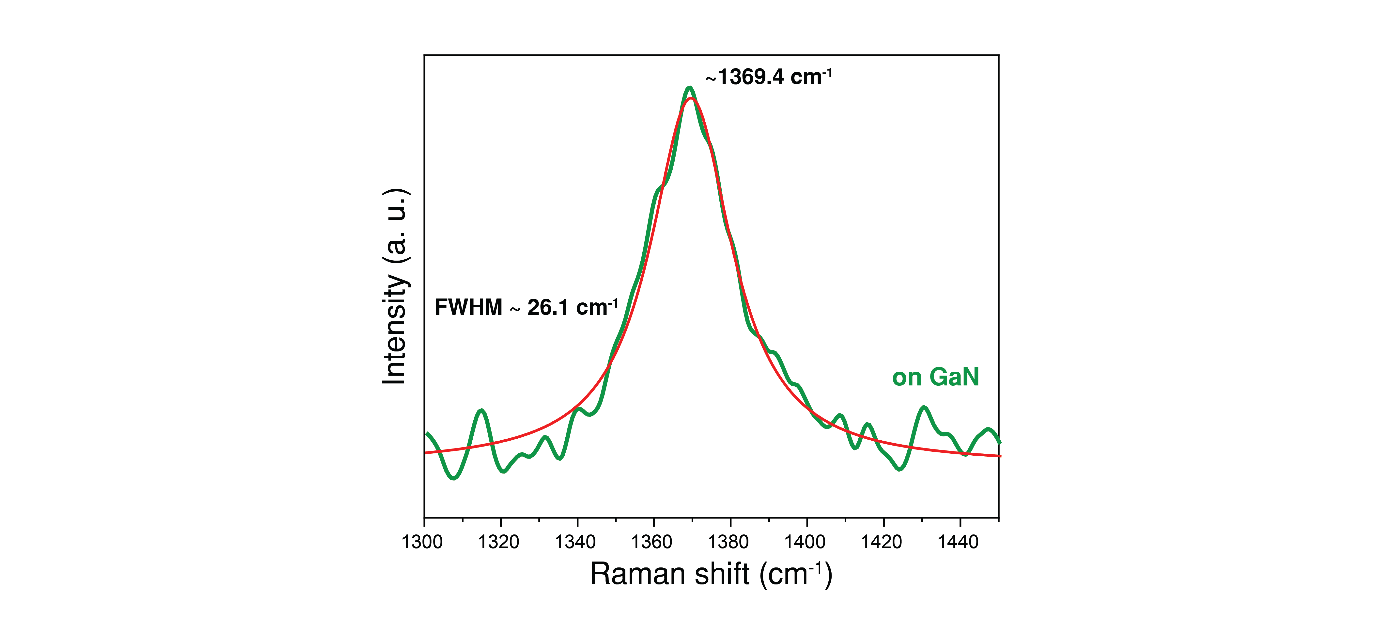


**Figure S5**. Raman spectra of h-BN in the active area of memristors (Green). E_2g_ peak fitting (Red) with Pseudo-Voigt function.


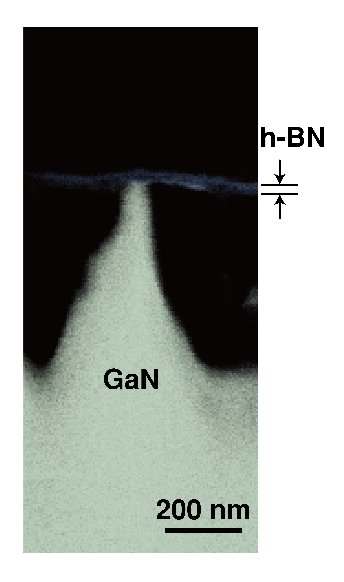


**Figure S6.** False-color low-angle annular dark-field STEM image of h-BN(H_2_) films supported by GaN nano-cones.


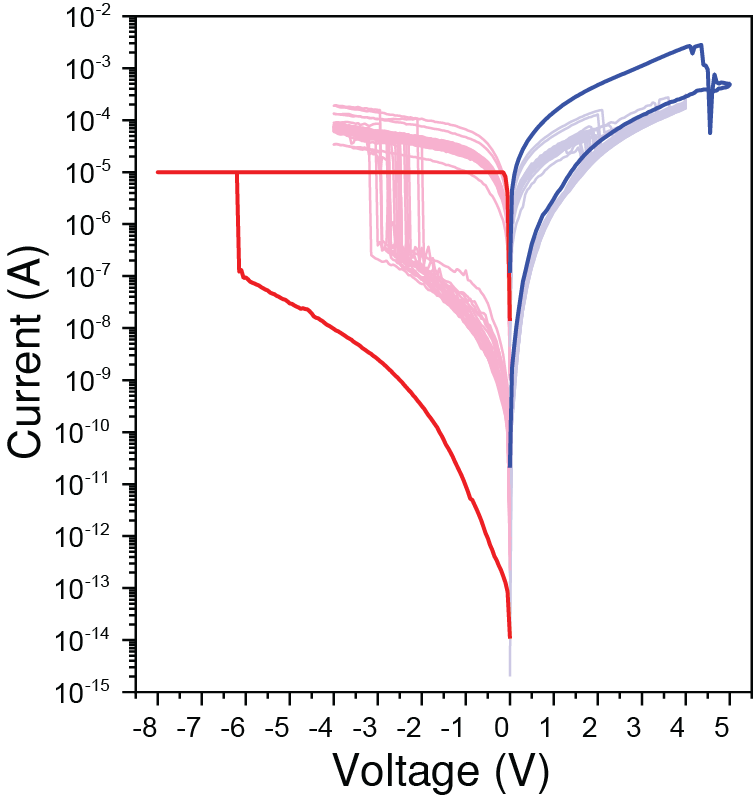


**Figure S7**. Electroforming process of a h-BN(N_2_) memristors. The first sweep is shown in red (negative sweep) and blue (positive sweep), while the subsequent sweeps are shown in light red and light blue, respectively.


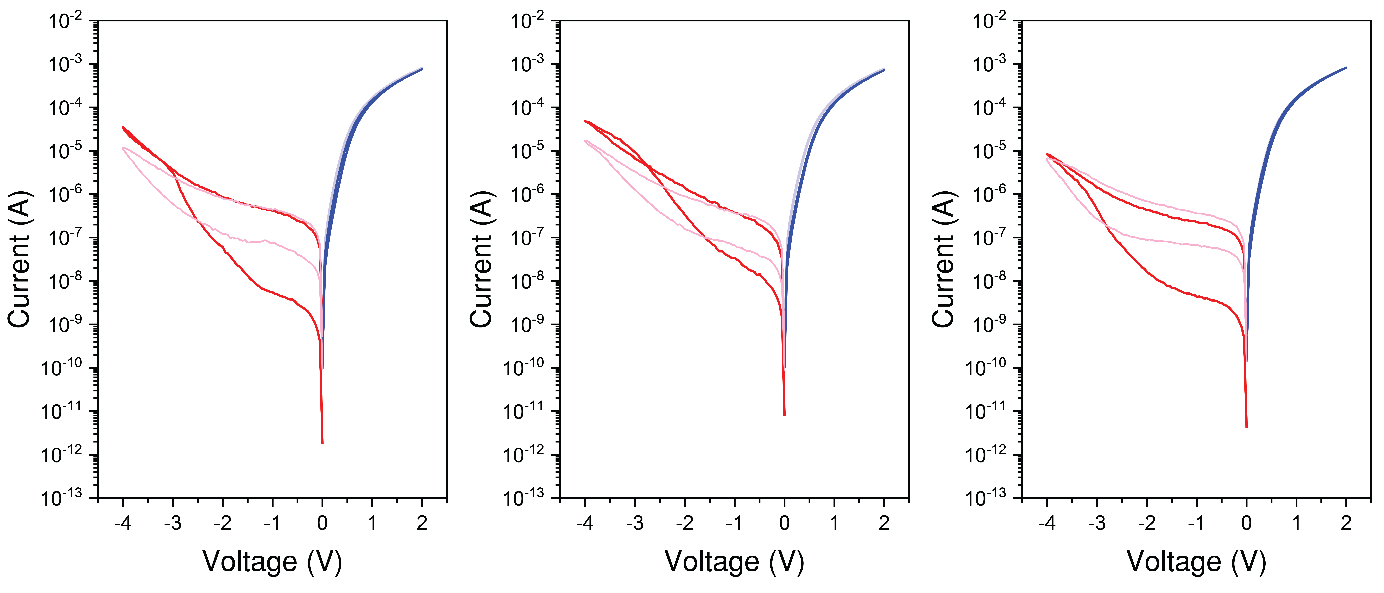


**Figure S8**. Electroforming process of three different h-BN(H_2_) memristors. The first sweep is shown in red (negative sweep) and blue (positive sweep), while the subsequent sweeps are shown in light red and light blue, respectively.


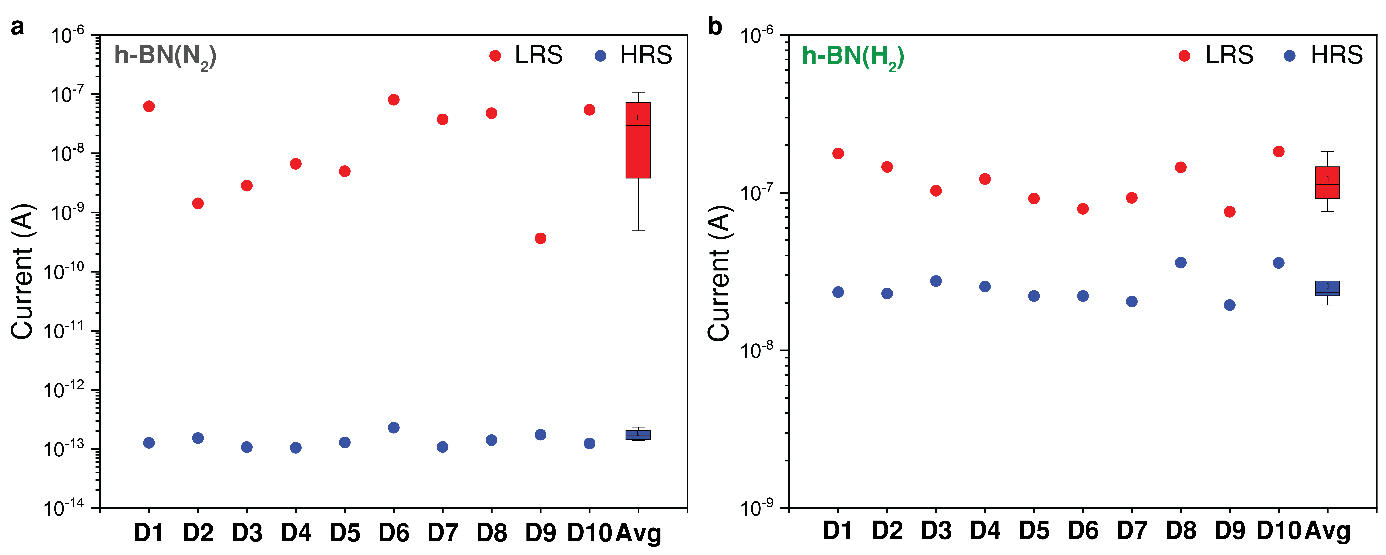


**Figure S9.** Statistical distribution of HRS and LRS current levels measured from (a) 10 h-BN(N_2_) memristors and (b) 10 h-BN(H_2_) memristors.


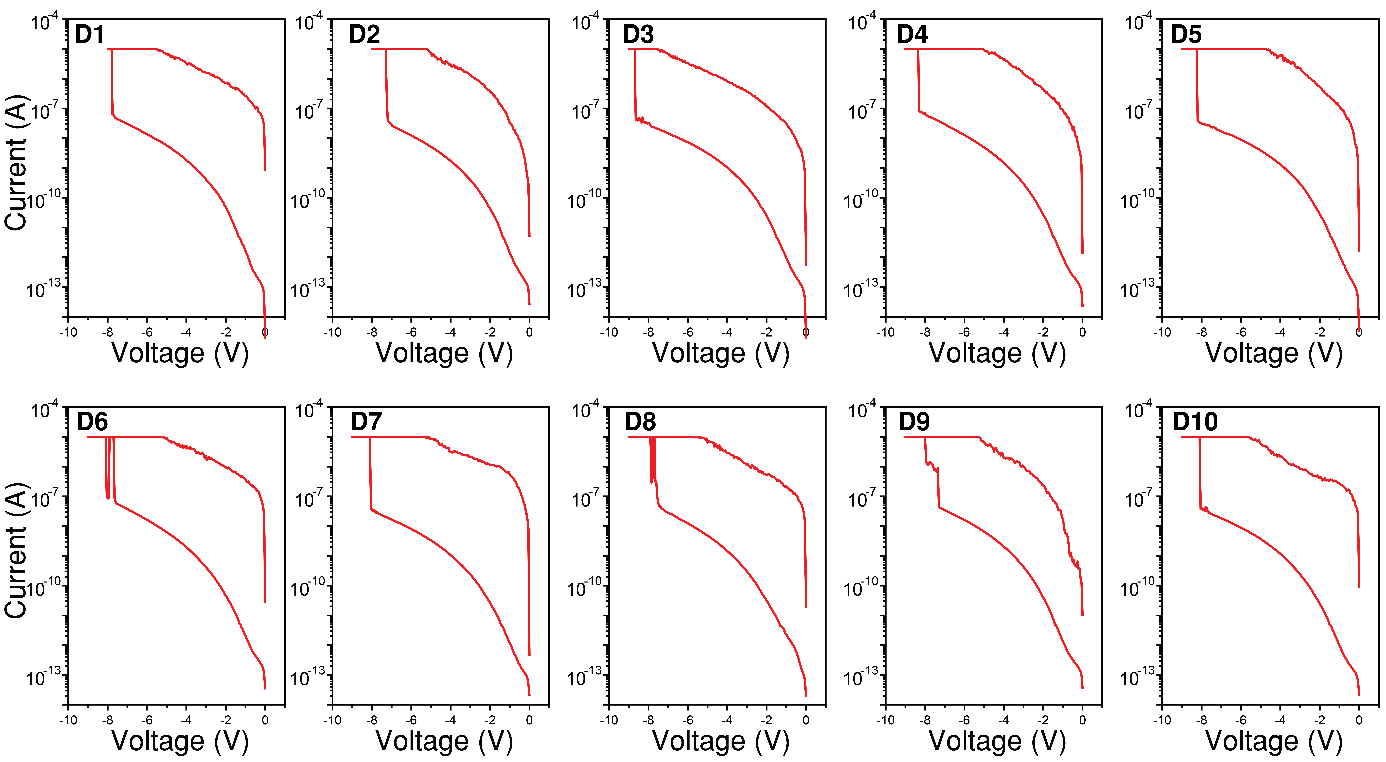


**Figure S10.** First-sweep I-V characteristics of 10 individual h-BN(N_2_) memristors.


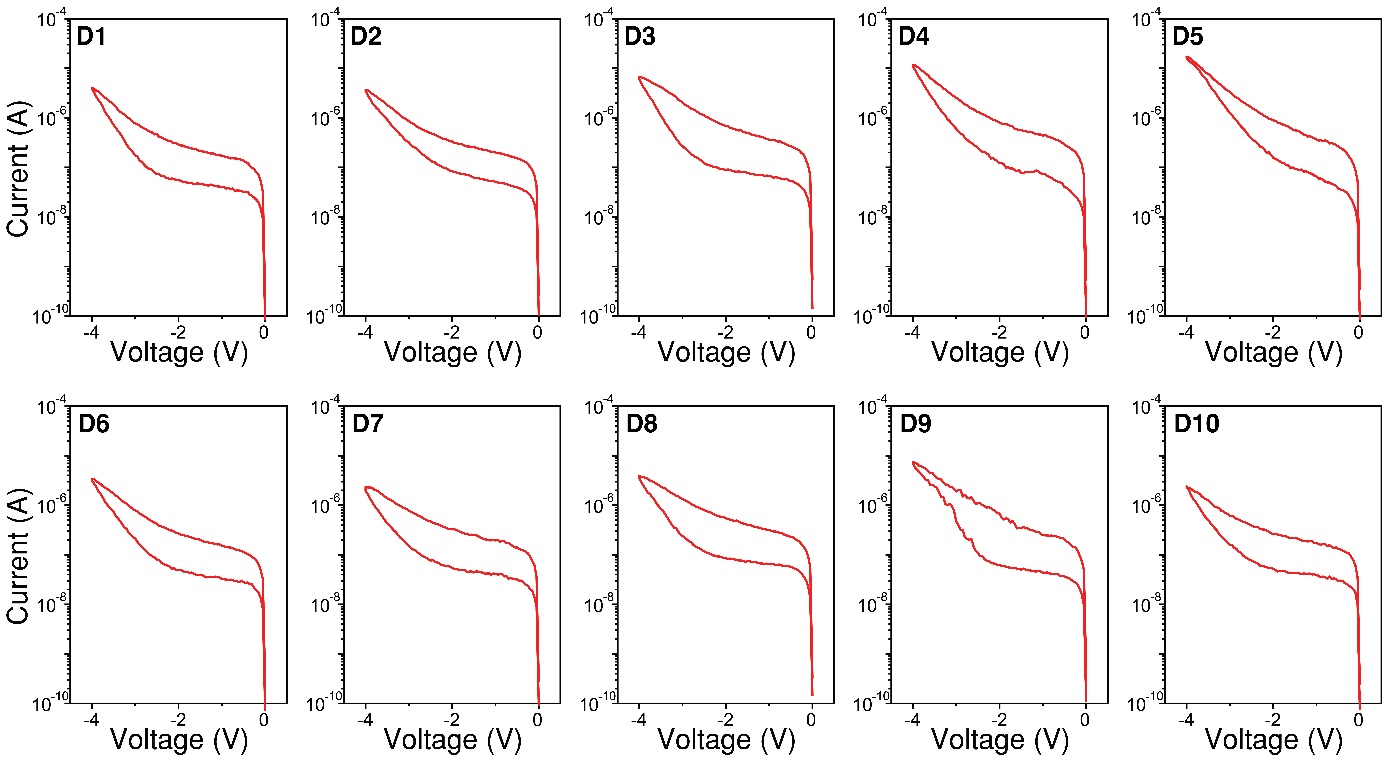


**Figure S11.** First-sweep I-V characteristics of 10 individual h-BN(H_2_) memristors.


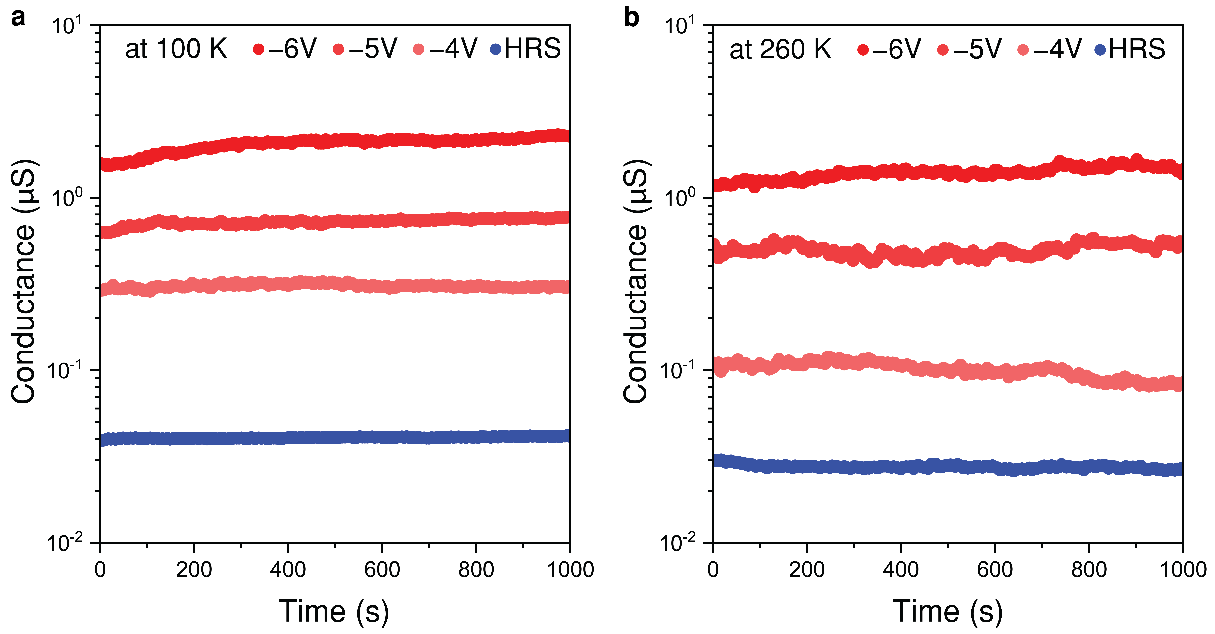


**Figure S12**. Retention characteristics of h-BN(H_2_) memristors measured over 1000 s at (a) 100 K and (b) 260 K, following SET operations at −4 V, −5 V, and −6 V.


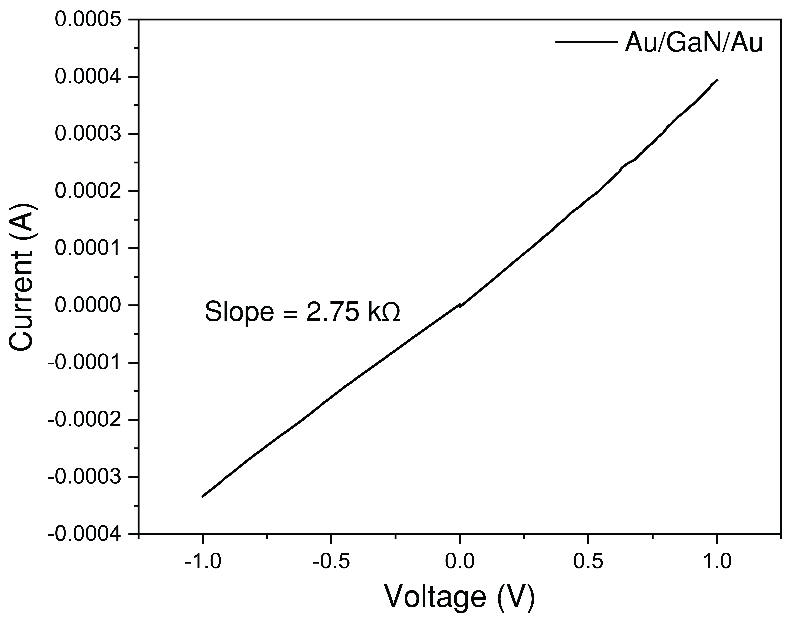


**Figure S13**. Linear I-V plot of a device with an Au/GaN/Au structure to evaluate the series resistance of the GaN bottom electrode used in the memristors.


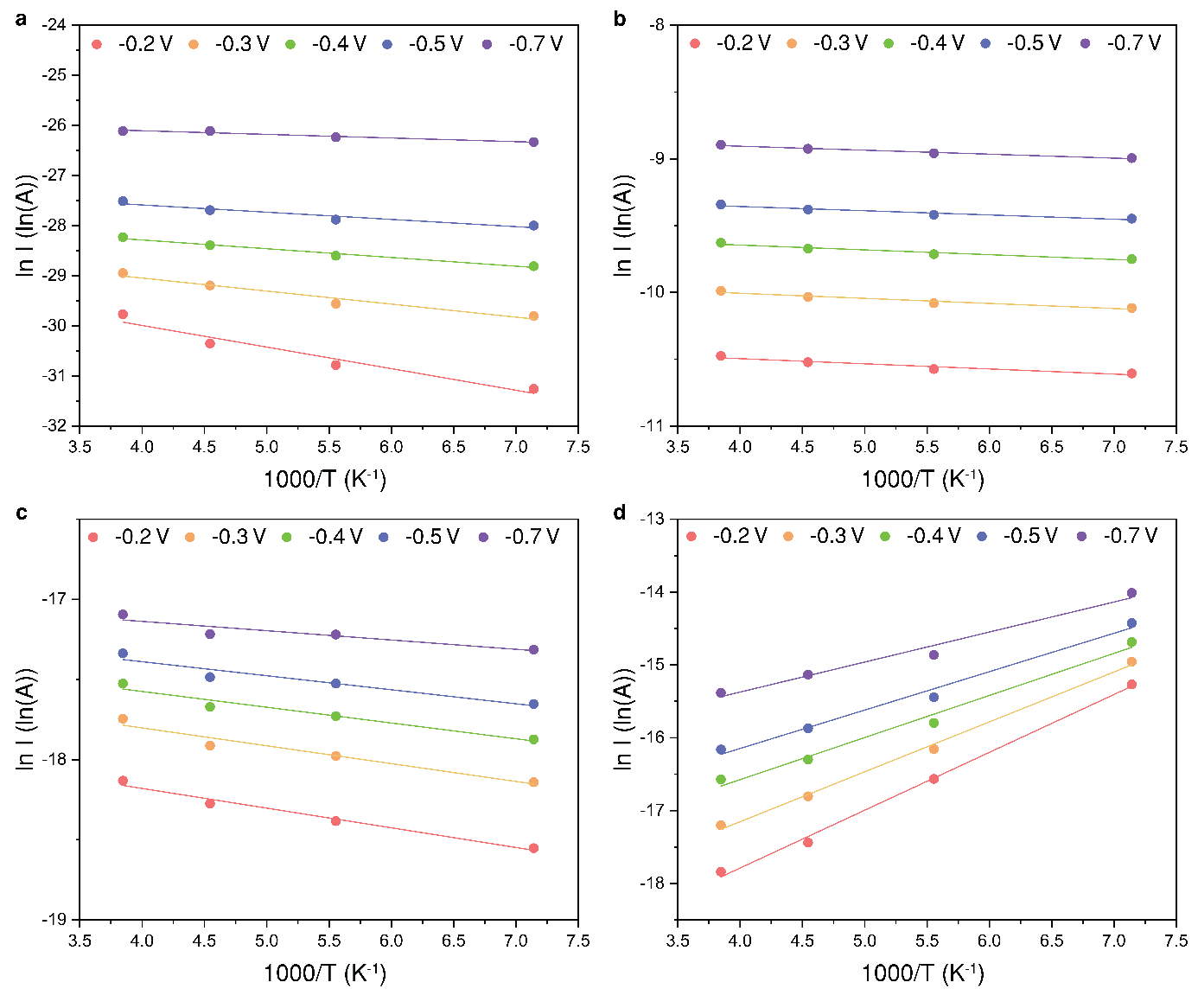


**Figure S14**. Arrhenius plots of ln I versus 1000/T for (a) the HRS of an h-BN(N_2_) memristor, (b) the LRS of an h-BN(N_2_) memristor, (c) the HRS of an h-BN(H_2_) memristor, and (d) the LRS of an h-BN(H_2_) memristor, measured at various voltages in the range of 140~260 K.


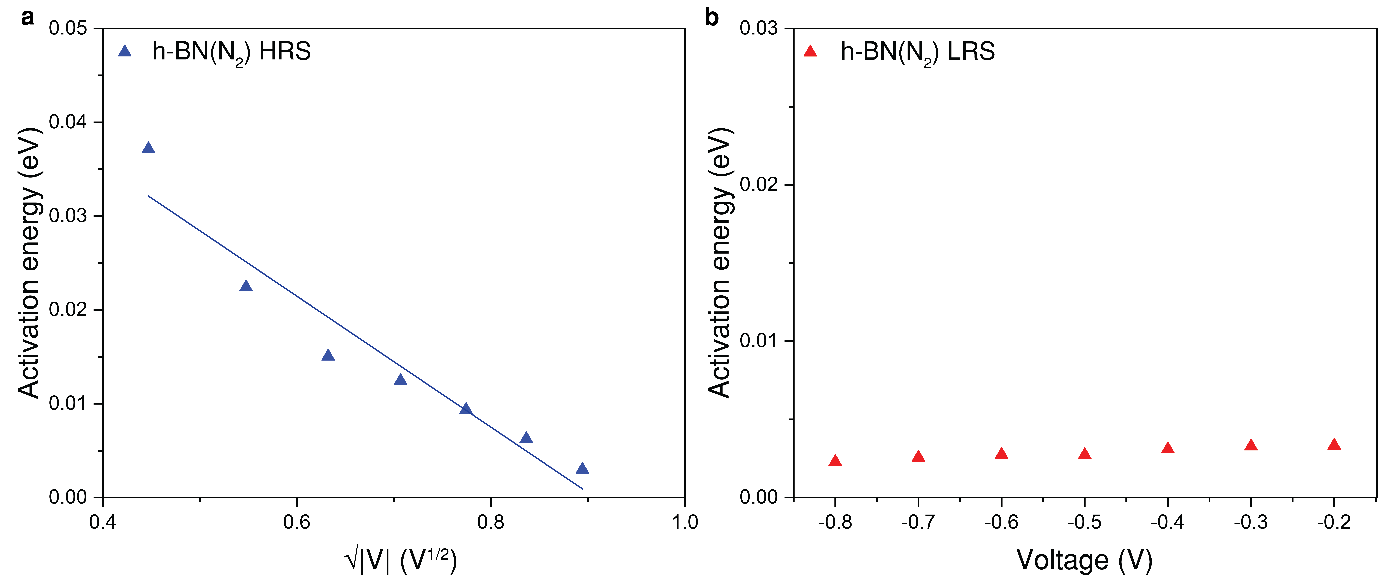


**Figure S15**. Activation energy of the h-BN(N_2_) memristor. (a) E_a_ extracted from Arrhenius plots in the HRS shows a linear relationship with √|V| (R^2^ = 0.93), consistent with a Poole-Frenkel-type emission mechanism. (b) In the LRS, E_a_ remains nearly constant at ~3 meV, indicating voltage-independent conduction through a stable filamentary channel.


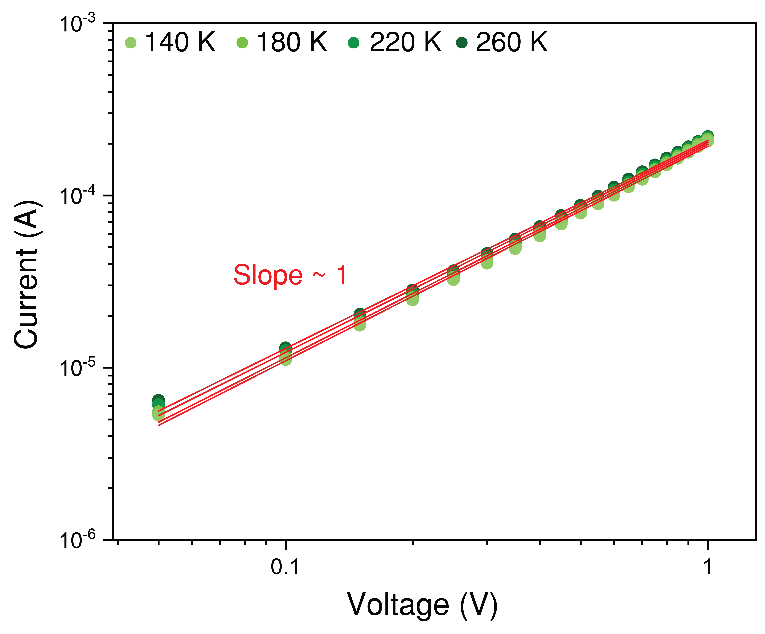


**Figure S16**. Double-logarithmic I-V characteristics at various temperatures (140~260 K), exhibiting Ohmic conduction (slope ~1)


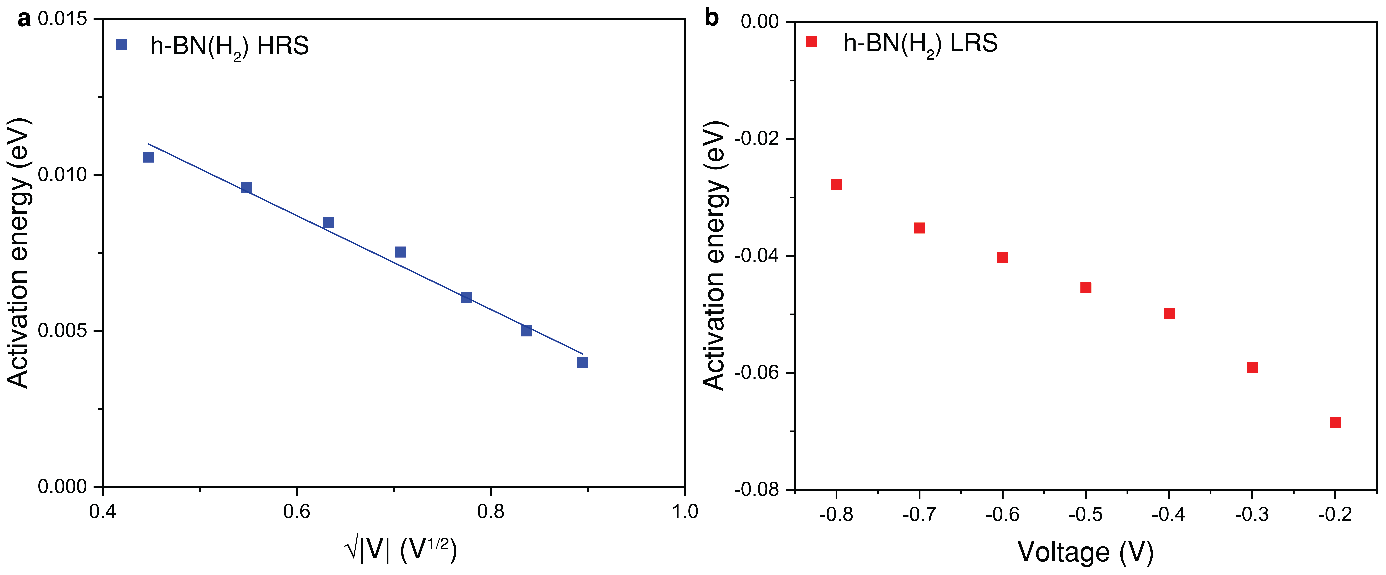


**Figure S17**. Activation energy extracted from the linear fits in Figure R19 as a function of voltage for (a) HRS of an h-BN(H_2_) memristor, (b) LRS of an h-BN(H_2_) memristor. The transition from positive in HRS to negative E_a_ in LRS indicates the formation of non-thermally active conduction paths.


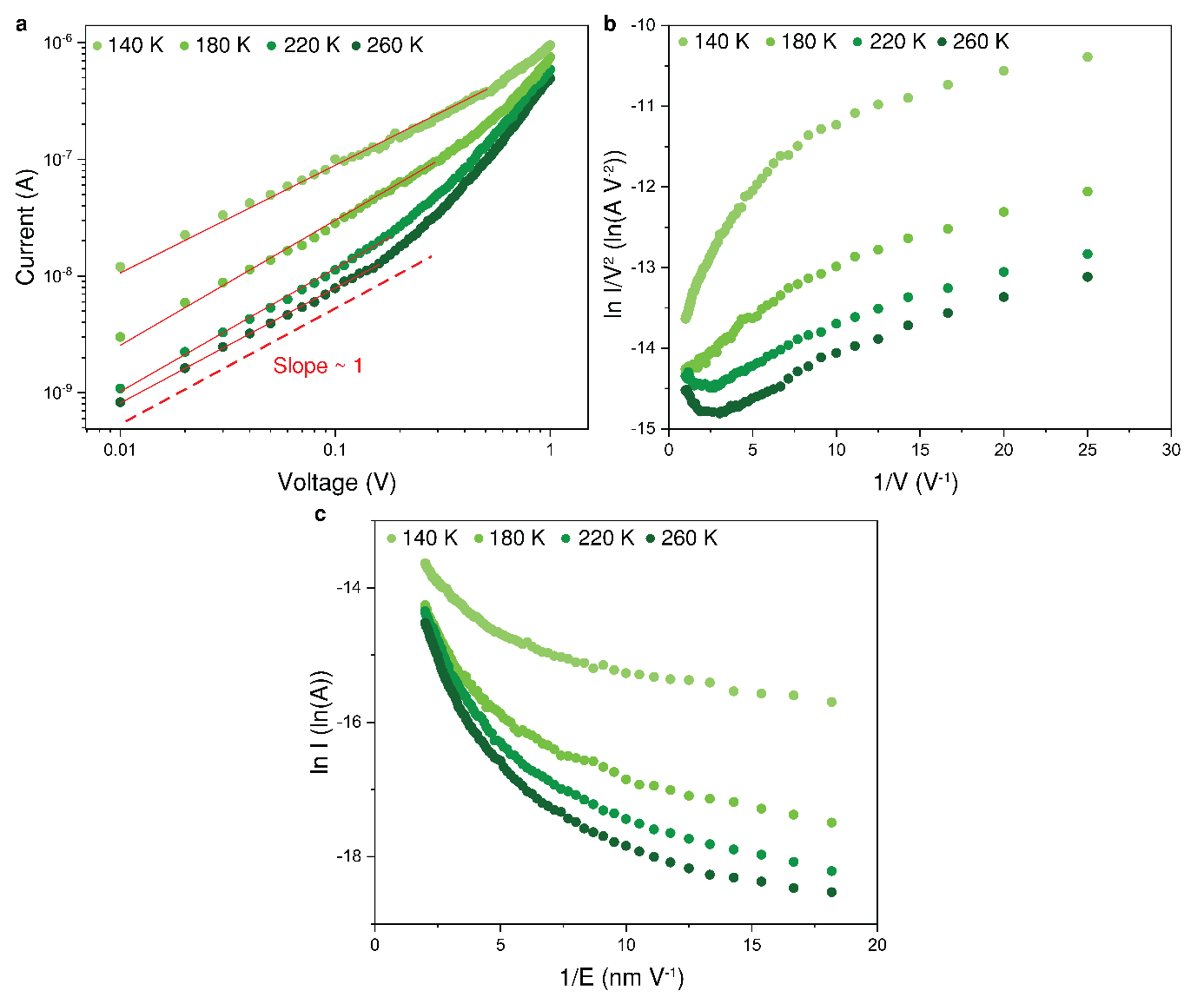


**Figure S18**. Temperature-dependent conduction analysis of the h-BN(H_2_) memristor in the LRS. (a) Double-logarithmic I-V curves. (b) Fowler–Nordheim plot, ln(I/V^2^) versus 1/V plot. (c) ln(I) versus 1/E plot.


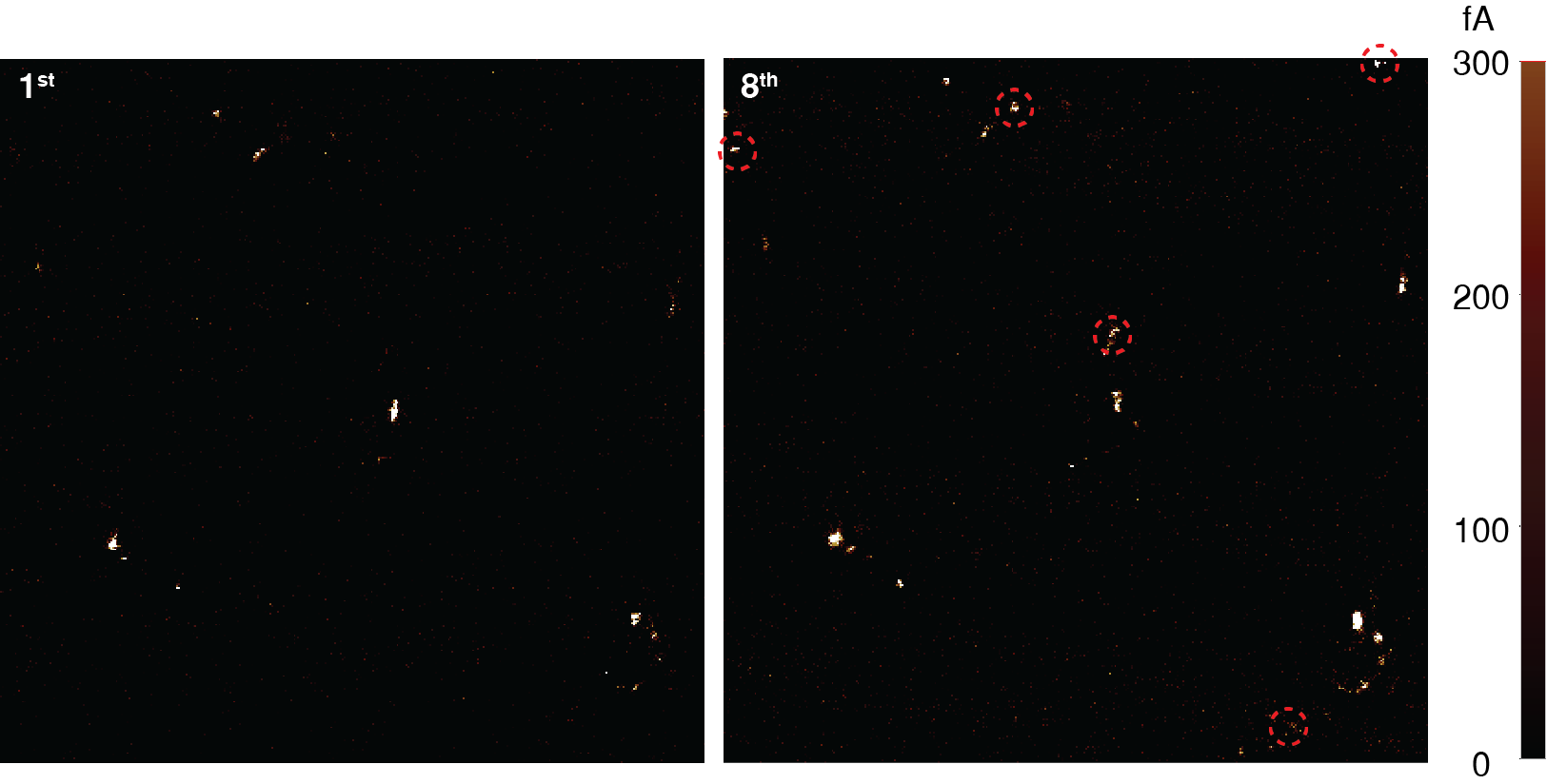


**Figure S19.** Formation of multiple nano-filaments observed at repeated current mappings under same bias condition of $-$6.5 V. The first mapping image (left) shows a smaller number of bright spots than the 8th mapping image (right). Red dotted circles represent the formation of new filaments after repeated mapping.


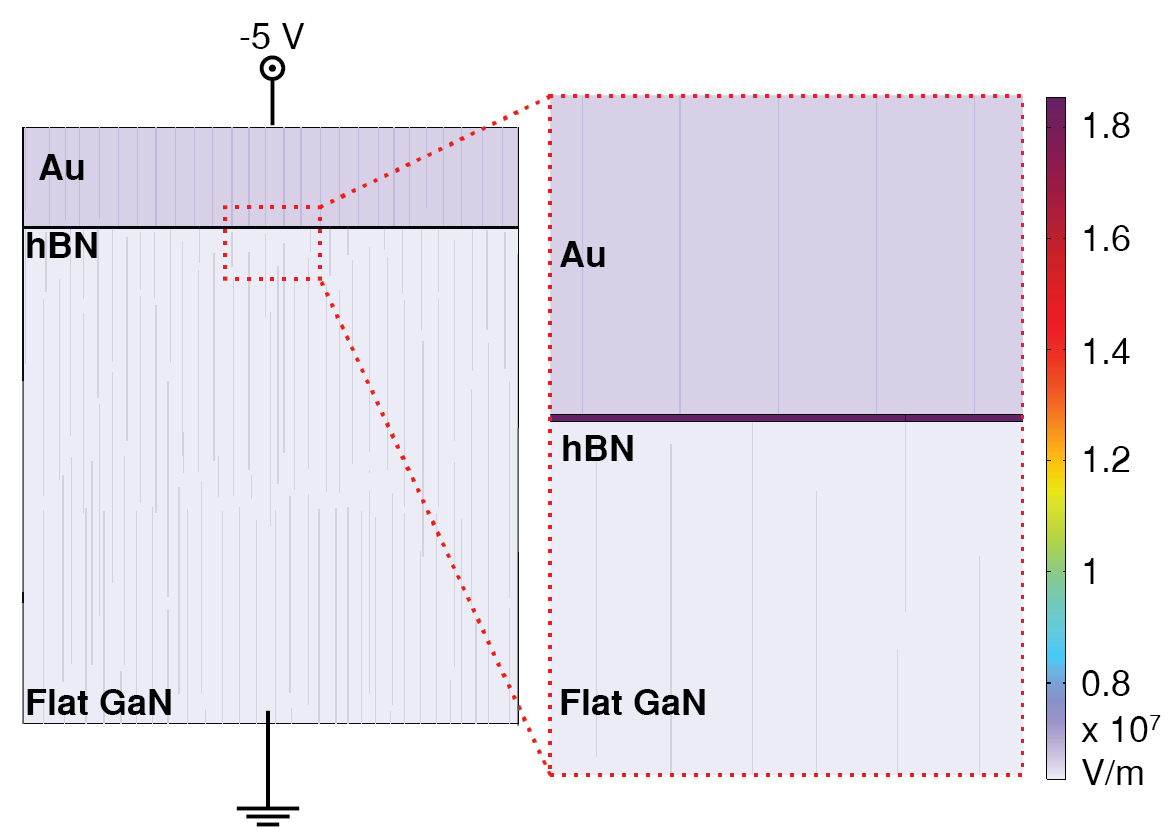


**Figure S20.** Electrical potential distributions of an Au/h-BN(N_2_)/GaN structured memristor during the SET process simulated by COMSOL Multiphysics simulation 6.0 under same conditions as those in Figure 3f.


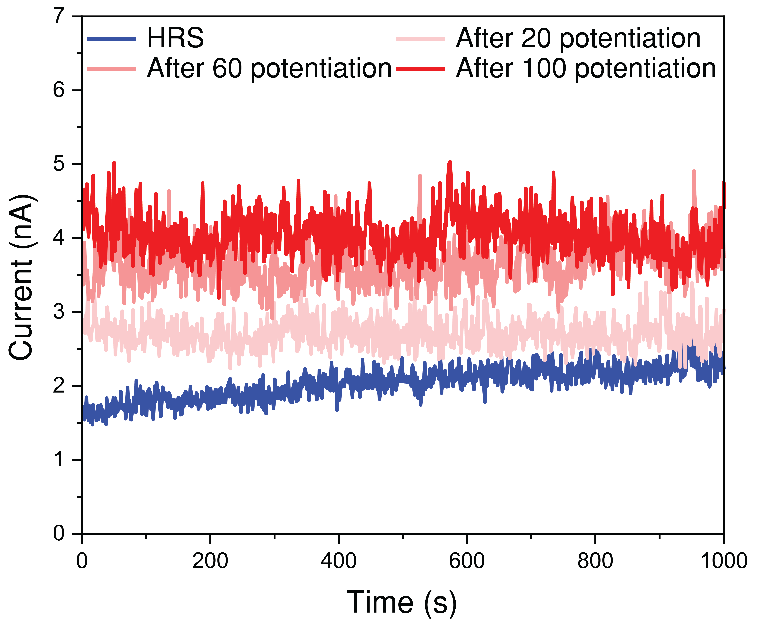


**Figure S21.** Retention characteristics of the h-BN(H_2_) memristor after applying 20, 60, and 100 potentiation pulses. Current levels were maintained for 1000 s after each potentiation level under a read voltage of −0.2 V.


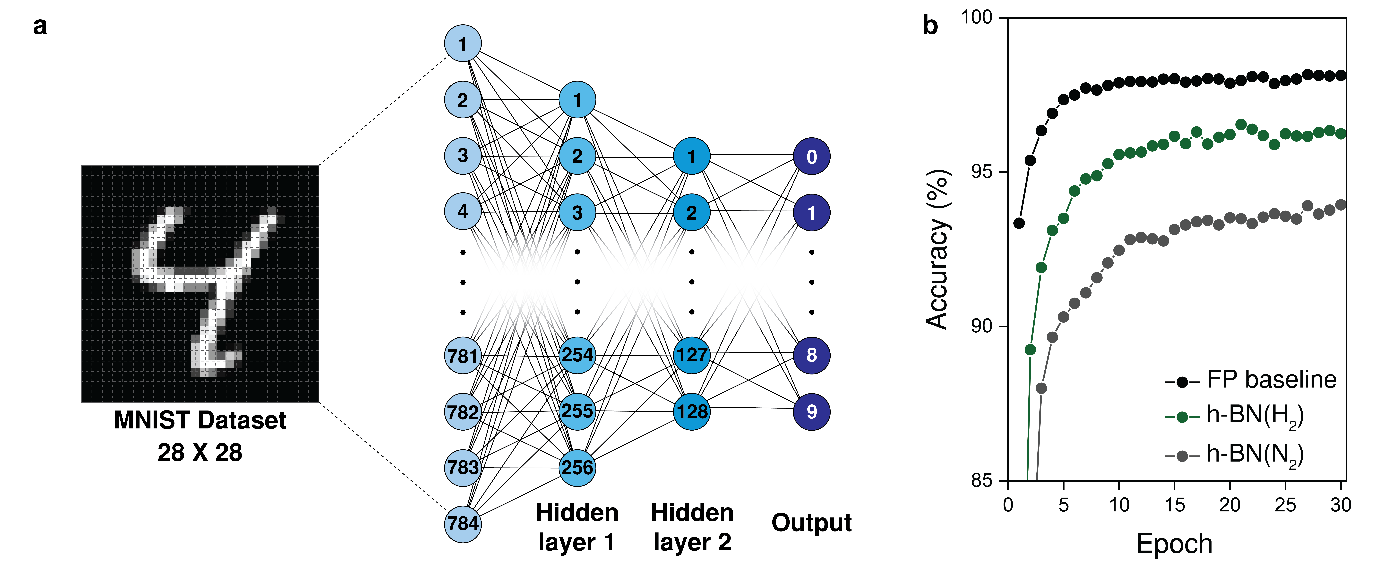


**Figure S22.** (a) Schematic illustration of a three-layer fully connected network structure for MNIST handwritten digit classification tasks (b) Accuracy of a FP baseline (black line), the h-BN(H_2_) memristor (green line), and the h-BN(N_2_) memristor (gray line) over 30 epochs, considering a 10% variation for both memristors.
